# Photodynamic therapy simultaneously induces ferroptosis- and apoptosis-like lipid signatures in ovarian cancer cells

**DOI:** 10.1038/s41419-025-08189-5

**Published:** 2025-12-10

**Authors:** Marta Overchuk, Albert M. Choi, Brittany P. Rickard, Brian Schnoor, Brandie M. Ehrmann, Albert W. Girotti, David A. Zaharoff, Huang-Chiao Huang, Thomas M. O’Connell, Shawn M. Gomez, Imran Rizvi

**Affiliations:** 1 Lampe Joint Department of Biomedical Engineering, University of North Carolina at Chapel Hill, Chapel Hill, NC, USA and North Carolina State University, Raleigh, NC USA; 2 Department of Biological Sciences, North Carolina State University, Raleigh, NC USA; 3 Curriculum in Toxicology & Environmental Medicine, University of North Carolina School of Medicine, University of North Carolina at Chapel Hill, Chapel Hill, NC USA; 4 Fischell Department of Bioengineering, University of Maryland, College Park, MD USA; 5 Department of Chemistry, University of North Carolina at Chapel Hill, Chapel Hill, NC USA; 6 Department of Biochemistry, Medical College of Wisconsin, Milwaukee, WI USA; 7 Lineberger Comprehensive Cancer Center, University of North Carolina School of Medicine, Chapel Hill, NC USA; 8 Marlene and Stewart Greenebaum Cancer Center, University of Maryland School of Medicine, Baltimore, MD USA; 9 Department of Otolaryngology, Head and Neck Surgery, Indiana University School of Medicine, Indianapolis, IN USA; 10 Department of Pharmacology, University of North Carolina at Chapel Hill, Chapel Hill, NC USA

**Keywords:** Ovarian cancer, Lipidomics

## Abstract

Resistance to apoptosis-inducing chemotherapy is a major factor contributing to treatment failure and poor survival outcomes in high-grade serous ovarian cancer (HGSOC). Ferroptosis, a regulated form of cell death driven by lipid peroxidation, has emerged as a promising effector mechanism because it remains available in HGSOC cells with impaired apoptosis signaling. While most research has focused on pharmacological ferroptosis inducers, there is growing interest in strategies that could trigger lipid autoxidation through externally delivered energy, such as photons. Photodynamic therapy (PDT), which utilizes light and light-activatable photosensitizers to generate reactive molecular species, offers a means of initiating lipid peroxidation with a high degree of precision and minimal systemic toxicities. However, the precise lipid targets of PDT, the influence of varying tumor lipidomic landscapes, and the role of ferroptosis sensitivity on PDT-lipid interactions have yet to be elucidated. In this study, we systematically compare PDT to ferroptosis induced by the inhibition of glutathione peroxidase 4, focusing on lipid redox states and composition in HGSOC cell lines. While PDT was similarly effective in both ferroptosis-sensitive and -resistant cells, its effects on cellular lipidomes differed markedly. PDT robustly induced lipid radical formation in both cell types; however, a dose-dependent accumulation of lipid hydroxides and hydroperoxides was only observed in ferroptosis-sensitive cells rich in unsaturated phospholipids. Further analysis revealed a significant overlap in lipid oxidation targets between PDT and ferroptosis. Notably, in both cell types, and in vivo, PDT upregulated ceramides, a lipid class strongly associated with mitochondrial apoptosis. In summary, PDT exhibited comparable efficacy in both ferroptosis-sensitive and -resistant cells by triggering a combination of lipid peroxidation and ceramide upregulation, suggesting the activation of both ferroptosis and apoptosis pathways. Further studies are needed to explore the role of PDT-induced lipidomic changes in the initiation of various cell death pathways and in overcoming chemoresistance in HGSOC.

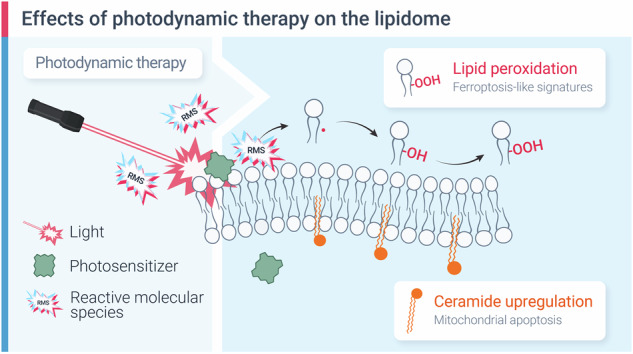

## Introduction

Despite considerable progress in understanding the origin and biology of high-grade serous ovarian cancer (HGSOC) [1, 2], the lack of reliable screening tools and pervasive resistance to standard-of-care chemotherapy are associated with a dismal 5-year survival rate of approximately 30% for advanced HGSOC [3–5]. Resistance to apoptosis has been identified as a major driver of chemotherapy failure, prompting significant interest in strategies that could simultaneously activate multiple cell death pathways, thereby synergistically targeting non-overlapping compensatory axes [6, 7]. Ferroptosis, an iron-dependent form of regulated cell death driven by lipid peroxidation, has emerged as a promising non-apoptotic effector mechanism due to its effectiveness in chemoresistant HGSOC [8–13].

Recent studies have shown that chemoresistant HGSOC cells often acquire a set of metabolic characteristics that render them sensitive to ferroptosis. These traits often include the accumulation of excess iron, which increases oxidative stress through the Fenton reaction [14], as well as alterations in the balance between saturated and unsaturated phospholipids [15–18]. Specifically, an increased proportion of unsaturated, and especially polyunsaturated, lipids in cell membranes makes them more vulnerable to oxidation [16, 19, 20]. Such metabolic rewiring results in an overreliance on glutathione peroxidase 4 (GPX4), the only mammalian enzyme capable of reducing lipid hydroperoxides to hydroxides, thereby preventing the propagation of oxidative membrane damage [9, 11, 21, 22]. Given its critical function in protecting cells against ferroptosis, most existing ferroptosis inducers either target GPX4 directly or promote the depletion of intracellular glutathione (GSH), which is essential for GPX4-mediated lipid hydroperoxide reduction.

Despite the significant interest in ferroptosis as a means of overcoming chemoresistance in HGSOC, its clinical translation remains challenging. Developing safe and effective small molecule inhibitors for GPX4 is challenging because of the broad substrate specificity and the relatively flat region surrounding the active site of the enzyme [23]. Most existing GPX4 inhibitors act covalently via a reactive alkyl chloride moiety, which confers poor selectivity and pharmacokinetic properties [24], as well as systemic toxicity [25]. Moreover, targeting the GPX4-GSH axis tends to only be effective in a subset of cells with already high ferroptosis sensitivity, meaning that ferroptosis inducers should not replace, but be used along with, conventional chemotherapy or other apoptosis-inducing agents [26]. Such combinations, however, could impose an additional toxicity burden on already grueling chemotherapy regimens. While efforts are underway to develop safer pharmacological ferroptosis inducers, complementary means of inducing ferroptosis, or a combination of ferroptosis and apoptosis, are in high demand.

Photodynamic therapy (PDT) is a photochemistry-based modality that relies on the activation of photosensitizers by light to locally generate cytotoxic reactive molecular species (RMS) [27, 28]. The advent of fiber optics and laparoscopic surgical techniques has enabled light delivery for imaging and PDT for most solid tumors and anatomical locations [28]. In patients with ovarian cancer, PDT has been successfully performed after cytoreductive surgery, where most macroscopic tumors are removed and the remaining non-resectable tumors on the peritoneal surfaces are exposed to light using a light diffuser [29–31]. Preclinical evidence increasingly suggests that low doses of PDT, or photodynamic priming, can sensitize tumor cells to adjuvant chemotherapy, helping to overcome both inherent and acquired chemoresistance [32–38]. Depending on the light dosimetry, physiochemical properties, and intracellular localization of the photosensitizer, the photochemical generation of RMS can induce a range of biological effects, from transient mitochondrial membrane depolarization and photodegradation of chemoresistance-associated drug efflux pumps to dramatic shifts in pro- and anti-apoptotic protein levels and cell death [39–41]. While the apoptosis-inducing effects of PDT are well-established, its capacity to induce lipid peroxidation and the broader impact on lipid composition remain less understood.

During PDT, a photosensitizer molecule absorbs a photon, leading to a series of electronic transitions [28]. The outcome of these transitions—either the generation of radicals (Type I reaction) or the formation of singlet oxygen through energy transfer (Type II reaction)—depends on the molecular structure of the photosensitizer and the local environment [42, 43]. Critically, if a photosensitizer is in close proximity to an unsaturated lipid molecule, this interaction can lead to the formation of a lipid radical or a lipid peroxide, thereby initiating a lipid peroxidation cascade [44, 45]. Indeed, recent studies suggest that PDT-induced cell death, under certain conditions, shares characteristics with ferroptosis [46, 47]. In an elegant study, Shui and colleagues demonstrated that PDT with hypericin can induce enzyme-independent lipid peroxidation, followed by a reduction in intracellular GSH levels [47]. Importantly, PDT-induced cell killing was equally effective in cells with and without acyl-CoA synthetase long chain family member 4 (ACSL4), an enzyme that incorporates oxidation-sensitive polyunsaturated fatty acids within the membrane and is essential in pharmacological ferroptosis [20]. While this study demonstrates the general importance of lipid peroxidation in photodynamic cell death, many questions remain with respect to PDT’s ability to induce ferroptosis-like cell death in cells with differing underlying lipid compositions and sensitivities to canonical ferroptosis inducers.

In this article, we compare the effects of PDT using benzoporphyrin derivative (BPD), a clinically-approved photosensitizer, and ML210, a small molecule ferroptosis inducer [48], on lipid composition, redox state, and relevant antioxidant systems in OVCAR-3 and Caov-3 cell lines that are representative of ferroptosis-sensitive and -resistant HGSOC, respectively. Prior studies, including our own, have demonstrated that BPD predominantly localizes to mitochondria and the endoplasmic reticulum (ER) [49, 50]. Given that RMS generated during PDT exhibits extremely short diffusion distances in biological environments [51], lipid radical generation is initiated within these intracellular compartments. While ML210 is only effective in ferroptosis-sensitive OVCAR-3 cells, BPD-enabled PDT maintains comparable efficacy in both cell lines. Leveraging liquid chromatography-mass spectrometry (LC-MS)-based lipidomics, this study identifies lipid oxidation targets affected by two mechanistically distinct approaches, revealing a significant overlap between ML210- and PDT-induced lipid oxidation targets in OVCAR-3, but not ferroptosis-resistant Caov-3 cells. In addition to lipid peroxidation, untargeted lipidomic analysis identifies global lipidome alterations induced by ML210 and PDT, highlighting the specific lipid classes and species modulated by each treatment modality. PDT-induced lipidomic changes, depending on the tumor cell phenotype, involve lipid peroxidation signatures consistent with ferroptosis as well as upregulation of ceramides (Cer) and cardiolipins associated with cytochrome c leakage and mitochondrial apoptosis [52, 53]. Upregulation of Cer is also observed in a subcutaneous OVCAR-3 mouse tumor model, accompanied by a significant decrease in ceramide-1-phosphate (Cer-1-P), a pro-survival Cer metabolite with anti-apoptotic properties [54]. To summarize, the ability of PDT to target various areas of the lipidome within the context of profound lipidomic heterogeneity in HGSOC highlights the value of this photochemistry-based approach for the treatment of advanced-stage disease.

## Materials & Methods

### Cell lines

OVCAR-3 and Caov-3 cells were obtained from the American Type Culture Collection (ATCC) and authenticated at the Vironomics Core at the University of North Carolina at Chapel Hill. OVCAR-3 cells were grown in RPMI 1640 medium (Gibco, Life Technologies, Grand Island, NY, USA) supplemented with 20% fetal bovine serum (FBS, HyClone, Logan, UT, USA, Cat No.: SH30396.03, Lot No.: AE25787266), 0.01 mg/mL recombinant human insulin (Gibco, Life Technologies, Grand Island, NY, USA), 100 U/mL penicillin, and 100 μg/mL streptomycin (Gibco, Life Technologies, Grand Island, NY, USA). Caov-3 cells were grown in Dulbecco’s Modified Eagle’s Medium High Glucose (DMEM, Sigma-Aldrich, St. Louis, MO, USA) supplemented with 10% FBS, 100 U/mL penicillin, and 100 μg/mL streptomycin. Cells were maintained in 2D monolayers at 37 °C in a humidified incubator with 5% CO_2_ and routinely tested for Mycoplasma contamination using the MycoAlert^TM^ PLUS Kit (Lonza Bioscience, Basel, Switzerland, Cat. No.: LT07-710). Cells were propagated until they reached passage 30, after which a fresh early-passage aliquot was thawed.

### Chemicals

ML210 (CAS No.: 1360705-96-9, ≥98% HPLC) was purchased from Sigma-Aldrich (St. Louis, MO). Stock solutions (5 mM) were prepared by dissolving ML210 powder in cell culture grade dimethylsulfoxide (DMSO) from ATCC, aliquoting, and storing at -80°C. Ferroptosis inhibitors, including Liproxstatin-1 (Lip-1, CAS No.: 950455-15-9, ≥98% HPLC, Sigma-Aldrich), Ferrostatin-1 (Fer-1, CAS No.: 347174-05-4, ≥95% HPLC, Sigma-Aldrich) and Deferoxamine mesylate salt (DFO, CAS No.: 138-14-7, pharmaceutical primary standard, Sigma-Aldrich) were dissolved in cell culture grade DMSO at 10 mM, aliquoted and stored at −80 °C. Fresh stock solutions of L-Buthionine-sulfoximine (BSO, CAS No.: 83730-53-4, ≥97% TLC, Sigma-Aldrich) were prepared each time in sterile phosphate-buffered saline without Ca^2+^ and Mg^2+^ ions (PBS) at 25 mM. Benzoporphyrin derivative (BPD, CAS No.: 129497-78-5, ≥94% HPLC, Sigma-Aldrich) stock solutions ( ~ 500 μM) were prepared in cell culture grade DMSO, aliquoted, and stored at −20 °C. BPD concentration in each aliquot was confirmed prior to use via UV-Vis spectrometry. C11 BODIPY (BODIPY 581/591 C11, Invitrogen, Cat. No.: D3861) stock solution was prepared in DMSO at 2 mM, aliquoted, and stored at −20 °C. Dosing solutions were prepared in complete cell culture media immediately before treatments unless specified otherwise.

### Cell treatments

OVCAR-3 and Caov-3 cells were trypsinized, counted, and seeded in white-walled clear-bottom 96-well plates (Corning, Cat. No.: 3903) at 1 × 10^4^ cells per well unless otherwise specified. After 48 h, media was removed, and cells were treated with ML210 dosing solutions freshly prepared from DMSO stocks. Each experiment included a vehicle control group treated with DMSO v/v% corresponding to the highest ML210 concentration. After 72 h, survival fractions were determined using CellTiter-Glo assay as described below.

For mitochondria- and ER-targeting PDT (conventional protocol), cells were incubated with 0.25 μM BPD in complete culture media for 90 min. Following incubation, BPD-containing media were replaced with fresh media, and cells were irradiated with 690 nm light (LEDBox, BioLambda) at 20.04 mW/cm^2^. For short drug-light interval PDT, media were replaced with 2.5 μM BPD-containing media immediately before light exposure. Light doses were delivered using a programmable controller (BlackBox Smart, BioLambda), which automatically calculated irradiation times to achieve the desired energy densities. Each PDT experiment included a photosensitizer-only control group (BPD dark): 0.25 μM BPD for 90 min (conventional PDT protocol) or 2.5 μM BPD for the duration of light exposure (short drug-light interval protocol), without light irradiation.

### Inhibition studies

For pharmacologic ferroptosis inhibition experiments, OVCAR-3 and Caov-3 cells were co-incubated with ML210 and either 2.5 μM Lip-1, 2.5 μM Fer-1, or 1.25 μM DFO. For conventional PDT ± inhibition experiments, OVCAR-3 and Caov-3 cells were co-incubated with 0.25 μM BPD and either 10 μM Lip-1 or 25 μM DFO for 90 min. The dosing solutions were then replaced with fresh complete cell culture media, and cells were irradiated with 690 nm light at the indicated energy densities. For short drug-light interval PDT, cells were pre-incubated with 10 μM Lip-1 or 25 μM DFO for 90 min prior to the addition of 2.5 μM BPD and light irradiation. Lipid radical formation was assessed at 24 h using the C11 BODIPY plate reader assay, and survival fractions were determined at 72 h using the CellTiter-Glo assay.

### Assessment of survival fractions

After respective treatments, cells in white-walled, clear-bottom 96-well plates were subjected to CellTiter-Glo Luminescent Cell Viability Assay (Promega Corp., Madison, WI, Cat. No.: G7572) as per manufacturer’s instructions. Briefly, plates were removed from the incubator and allowed to equilibrate to room temperature for 30 min. During this time, the media was removed and replaced with 50 μL of fresh media. Next, 50 μL of CellTiter-Glo reagent was added in each well. Prior to reading luminescence, plates were shaken for 2 min at 80 rcf and then equilibrated for 10 min at room temperature. White tape was placed on the bottom of each plate to maximize the signal. Luminescence measurements were conducted using a CLARIOstar Plus plate reader (BMG LABTECH, Germany) with Enhanced Dynamic Range settings. Survival fractions were calculated by normalizing the luminescence value in each well to the average luminescence of control wells.

### GPX4 ELISA

OVCAR-3 and Caov-3 cells were seeded in 6-well plates at 3 × 10^5^ cells per well. After 48 h, cells were treated with 2.5 μM ML210 or PDT at energy densities of 0.05, 0.25, and 1 J/cm². Control conditions included media only, equivalent volumes of DMSO, and 0.25 μM BPD without light. Six hours post-treatment, cells were trypsinized, rinsed once with cold PBS, pelleted, and flash-frozen in liquid nitrogen. Prior to trypsinization, supernatants and PBS washes were collected in pre-labeled tubes and combined with the trypsinized cell pellets. This step ensured that cells detached from the surface were captured by the assay. GPX4 levels were measured using the Human Glutathione Peroxidase 4 ELISA Kit (Abcam, Cat. No.: ab304936). Briefly, cell pellets were lysed in 1X Cell Extraction Buffer containing Halt™ Protease Inhibitor Cocktail (Thermo Scientific™, Cat. No.: 87786). Protein concentration was determined using the Pierce™ BCA Protein Assay Kit (Thermo Scientific, Cat. No.: 23227), and lysates were diluted to 0.01 mg/mL with Sample Diluent Buffer. Fifty microliters of pre-diluted sample were mixed with 50 μL of the antibody cocktail in the supplied microplate and incubated for 1 h at room temperature on a shaker. After incubation, supernatants were discarded, and the plate was washed three times with 1X Wash Buffer. The plate was then incubated with TMB Development Solution, and color development at 600 nm was monitored every 60 s using a CLARIOstar Plus plate reader. The reaction was stopped once the maximum O.D. values reached 1.0 using the stop solution, and the final O.D. was measured at 450 nm. GPX4 concentration was determined from a standard curve.

### GSH-Glo assay

Intracellular GSH levels were measured using the GSH-Glo kit from Promega (Cat. No.: V6911). Briefly, OVCAR-3 and Caov-3 cells were seeded in white-walled, clear-bottom 96-well plates at 1 × 10^4^ cells/well, incubated for 48 h, and then treated with 2.5 μM ML210 and PDT at 0.05, 0.25, and 1 J/cm^2^. Control conditions included media only, equivalent volumes of DMSO, 0.25 μM BPD without light, and BSO at 500 μM. Six hours later, plates were centrifuged at 200 rcf for 5 min, supernatants removed and replaced with 100 μL of 1X GSH-Glo™ Reagent, agitated for 5 s, and incubated at room temperature for 30 min. Next, 100 μL of reconstituted Luciferin Detection Reagent was added. The plate was agitated for 5 s and allowed to equilibrate for another 15 min. White tape was placed on the bottom of each plate to maximize the signal, and luminescence measurements were conducted using a CLARIOstar Plus plate reader. Relative GSH content was determined by normalizing the luminescence value in each well to the average luminescence of control (media only) wells.

### C11 BODIPY plate reader assay

OVCAR-3 and Caov-3 cells were seeded in white-walled, clear-bottom 96-well plates at 1 × 10^4^ cells per well (3.13 × 10^5^ cells/cm^2^ of growth area) and allowed to grow for 48 h before any treatment was administered. At -24, -6, -3 and -1.5 h time points, cells were exposed to ML210 (2.5 μM) or PDT (0.25 J/cm^2^). For PDT-treated cells, 0.25 μM BPD was administered 1.5 h before irradiation, and photosensitizer-containing media was replaced with fresh complete cell culture media immediately prior to irradiation. At time 0, plates were centrifuged at 200 rcf for 5 min to sediment any floating cells, media was gently removed and replaced with FBS-free media containing 10 μM C11 BODIPY. Plates were incubated with the dye for 30 min, after which C11 BODIPY-containing media was gently rinsed with Hank’s Balanced Salt Solution (HBSS) once. Oxidized (excitation: 480/10 nm, emission: 530/20 nm) and unoxidized (excitation: 560/15 nm, emission: 600/15 nm) C11 BODIPY fluorescence in each well was measured using a CLARIOstar Plus plate reader. Oxidized/Total C11 BODIPY fluorescence ratios were calculated and normalized to control wells in the same plate for each cell line.

### C11 BODIPY confocal microscopy

OVCAR-3 and Caov-3 cells were seeded in 8-well microscopy chambers (Ibidi GmbH, Germany, Cat. No.: 80826) at 31.25 k cells/cm^2^ of growth area and allowed to grow for two days. Cells were exposed to 0.25 μM ML210 or DMSO, incubated for 6 h, stained with 10 μM C11 BODIPY in FBS-free media, rinsed once in HBSS, and imaged using FV3000 Confocal Laser Scanning Microscope (Olympus, Tokyo, Japan) at 20x magnification. For OVCAR-3 cells, the following imaging settings were used: oxidized C11 BODIPY: excitation: 488 nm, emission: 500-550 nm, laser power: 7%, PMT voltage: 720; unoxidized C11 BODIPY: excitation: 561 nm, emission: 570-610 nm, laser power: 1%, PMT voltage: 510. For Caov-3 cells, although identical oxidized C11 BODIPY imaging settings were used, PMT settings for unoxidized C11 BODIPY had to be increased to 525 to achieve comparable dynamic range.

To visualize lipid radicals generated due to chemical oxidation, cells were pre-stained with 10 μM C11 BODIPY in FBS-free media, after which C11 BODIPY-containing media was replaced with FBS-free media containing 50 μM cumene hydroperoxide (Cum OOH). After 1.5 h, Cum OOH was removed, cells were counterstained with Hoechst dye (1 µg/mL), and imaged. To visualize in situ lipid radical formation upon photodynamic activation, cells were first incubated with 0.25 μM BPD for 1 h, which was then replaced with FBS-free medium containing 0.25 μM BPD and 10 μM C11 BODIPY for 0.5 h. BPD and C11 BODIPY-containing media were then removed. Cells were rinsed once with HBSS and replaced with fresh cell culture medium. Next, each field of view was pre-imaged to collect baseline oxidized and unoxidized C11 BODIPY fluorescence, irradiated for 15–90 s with 405 nm confocal laser at 5%, and immediately imaged again to capture changes in oxidized and unoxidized C11 BODIPY fluorescence. Integrated densities for each image were measured in ImageJ, oxidized C11 BODIPY fluorescence was normalized by oxidized + unoxidized fluorescence.

### Caspase-Glo 3/7 assay

OVCAR-3 and Caov-3 cells were seeded in white-walled, clear-bottom 96-well plates at 1 × 10^4^ cells/well and cultured for 48 h. Cells were then treated with ML210 (2.5 μM), PDT (0.25 J/cm²), or DMSO (vehicle control). Six hours later, caspase-3/7 activity was measured using the Caspase-Glo 3/7 Assay (Cat. No. G8091, Promega) according to the manufacturer’s instructions. Briefly, the Caspase-Glo substrate and buffer were thawed, mixed, and stored at 4 °C for up to two weeks. Treatment media were removed and replaced with 100 μL fresh media, followed by 100 μL of Caspase-Glo 3/7 reagent per well, taking care to avoid cross-contamination. Plates were sealed, gently mixed at 80 rcf for 30 s, and incubated at room temperature for 30 min. Luminescence was recorded using a CLARIOstar Plus plate reader. Background luminescence (from media + reagent-only wells without cells) was subtracted from each sample. Resulting values were normalized to control wells on the same plate treated with media alone.

### OVCAR-3 tumor xenograft study

All animal studies were carried out according to the protocol approved by the Institutional Animal Care and Use Committee (IACUC) at the University of Maryland, College Park (Protocol R-MAR-25-09). OVCAR-3 cells were cultured in 120 mm diameter dishes (Thermo Scientific, Cat. No.: 168381) using the complete RPMI 1640 media as described above. Once the OVCAR-3 cells reached confluency, they were harvested using 0.25% Trypsin, 2.21 mM EDTA (Corning, Cat. No.: 25-053-CI) and rinsed with PBS. Cells were then resuspended in sterile saline solution at a concentration of 8.3 × 10^4^ cells/μL. Immunocompromised J:NU mice (Jackson Laboratory, Homozygous #007850, 8 weeks old) were anesthetized with 2–4% Isoflurane. Then, 60 μL of the cell solution, for a total of 5 × 10^6^ cells, were injected subcutaneously into each flank using a 28 G insulin syringe (Brandzig, Cat. No.: CMD2624). The flank tumors were then monitored using caliper measurements taken three times weekly. The volume of the subcutaneous tumor was estimated by multiplying the major axis by the square of the minor axis and dividing by 2.

Once the average tumor volume between left and right tumors reached approximately 70 mm^3^ (39.3 – 127.26 mm^3^; mean: 70.8 mm^3^), mice were randomly assigned to experimental groups with the consideration that the difference in average tumor volume does not exceed 10 mm^3^ between the treatment cohorts. Based on the very large Cer fold changes observed in vitro (>10-fold for some lipids), a sample size of n = 5 per group was selected, which corresponds to Cohen’s d ≈ 2.02 at α = 0.05 and 80% power (two-sample t test, two-sided, equal n, equal variances). BPD was administered in the form of NanoVP, a nanosuspension that enables BPD administration in aqueous solution described in Quinlan et al. [55, 56]. All mice in the BPD treatment group were given 1 mg/kg of NanoVP via a 50 μL tail vein injection under anesthesia. After a 90 min drug light interval, one flank tumor of the BPD-treated mouse was then topically irradiated with 690 nm wavelength light (Modulight 6600, ML6600-635-690) utilizing a light diffuser (ThorLabs, GBE05-B) suspended over the mouse flank. The mouse was treated at an irradiance of 100 mW/cm^2^ over a 25 min period for a total energy density of 150 J/cm^2^. During the procedure, the mouse was draped with blackout cloth to ensure that only the treated tumor site received the light dose (“PDT”). The contralateral tumor was not treated with light (“BPD”). Additionally, a control group of mice was treated with the same irradiance and light dose at one tumor location to study the effect of the light dose alone (“Laser”). Likewise, the contralateral tumor for these control mice was left untreated (“Control”). Six hours after the light treatment, the mouse was euthanized, and the subcutaneous tumor was collected. The tumor tissue was weighed, and then immediately snap-frozen in liquid nitrogen. All samples were stored at −80 °C and/or transported on dry ice prior to lipidomic analysis.

### Lipidomic workflows

OVCAR-3 and Caov-3 cells were seeded in 6-well plates at 3 × 10^5^ cells per well. After 48 h, the cells were treated with 2.5 μM ML210 or PDT at energy densities of 0.05, 0.25, and 1 J/cm². Control conditions included media only, equivalent volumes of DMSO, and 0.25 μM BPD without light. Six hours post-treatment, cells were trypsinized, rinsed once with cold PBS, pelleted, flash-frozen in liquid nitrogen, and stored at −80 °C. Methyl tert-butyl ether (MTBE), 1 mL, was added to cell pellets, vortexed, and then transferred to 1.5 mL centrifuge tubes. 300 µL of 1.5 μg/mL of Equisplash (Avanti Research) in methanol (internal standard) was added, and samples were shaken for 10 min. 200 µL of water was added to facilitate phase separation. The extracts were centrifuged at 20,000 rcf for 10 min. The top layer was removed, dried down, and reconstituted in 100 µL of isopropyl (IPA) alcohol for analysis. Analysis was performed using a Thermo Q Exactive HFX coupled to a Waters Acquity H-Class LC. A 100 mm × 2.1 mm, 1.7 µm Waters BEH C18 column was used for separations. The following mobile phases were used: A- 60/40 acetonitrile (ACN)/H_2_O, B- 90/10 IPA/ACN; both mobile phases had 10 mM ammonium formate and 0.1% formic acid. A flow rate of 0.2 mL/min was used. The starting composition was 40% B, which increased to 95% B at 12 min (held until 15 min), then 40% B at 15.1 and held for 5 min for re-equilibration. Samples were analyzed in positive/negative switching ionization mode with top 5 data-dependent fragmentation. Tumor tissues from OVCAR-3 xenografts were homogenized using a BeadBeater. Internal standards in methanol were added, followed by MBTE extraction and LC-MS analysis as described above.

### Lipidomic data analysis

Raw data was analyzed by LipidSearch software. Lipids were identified by MS2 fragmentation (mass error of precursor = 5 ppm, mass error of product = 8 ppm). The identifications were generated individually for each sample and then aligned by grouping the samples (OxPAPC=C, HF = S1, Con=S2). Normalization was performed using EquiSplash from Avanti.

In vitro: Out of 2602 lipid species automatically identified with LipidSearch, 638 adducts and 42 internal standards were removed. The remaining 1922 lipid species were manually reviewed in LipidSearch. Lipid species with poorly resolved chromatogram peaks were removed, leaving 594 unique lipid species and 79 oxidized lipid species (containing -OH or -OOH group) that passed manual review. In the resulting clean dataset, all non-numerical entries and zeros were removed, and missing values were imputed using Viime [57]. Missing Not at Random (MNAR) values defined as variables with any one group with more than or equal to 70% of missing data were imputed as 0, whereas Missing Completely at Random (MCAR) variables with all groups with less than or equal to 40% of missing data were imputed through the k-nearest neighbor (KNN) approach.

In vivo: Out of 5676 lipid species automatically identified by LipidSearch, 1481 adducts and 52 internal standards were removed. The remaining 4143 lipids were manually reviewed, and those with poorly resolved chromatographic peaks were excluded. This left 2121 unoxidized and 288 oxidized lipid species (containing -OH or -OOH groups) that passed review. To assess changes at the lipid class level, the sum of peak areas was calculated.

### Statistics and data visualization

All experiments were performed with three or more independent replicates containing at least two technical replicates as specified in figure captions. Unpaired t-tests, one-way, or two-way ANOVAs were employed as specified in figure captions using GraphPad Prism (version 10.4.0) or Graphmatik (version 0.3.2, 2025). Tukey, Dunnet, or Šidák corrections for multiple comparisons were used in one- way and two-way ANOVAs, respectively. All tests are 2-sided using an alpha level of 0.05. To generate volcano plots and heatmaps, fold changes, *p*-values, and z-scores were calculated, and plots were generated in R (version 4.4.1 2024-06-14) or Graphmatik. To analyze dose-response relationships, the null zone method was applied in R [58]. The gray zones in the plots represent the null zone, which is defined as the region where the 95% confidence intervals of the data points from each curve do not overlap. All samples were initially included in the analysis. Individual data points identified as significant outliers by Grubbs’ test (α = 0.05) were excluded. Given the small sample sizes, formal variance equality testing was not performed. Statistical methods assuming equal variances were applied as is conventional in preclinical exploratory research.

## Results

### OVCAR-3 and Caov-3 cells exhibit differences in sensitivity to chemical lipid peroxidation and phospholipid unsaturation

**Fig. 1:**
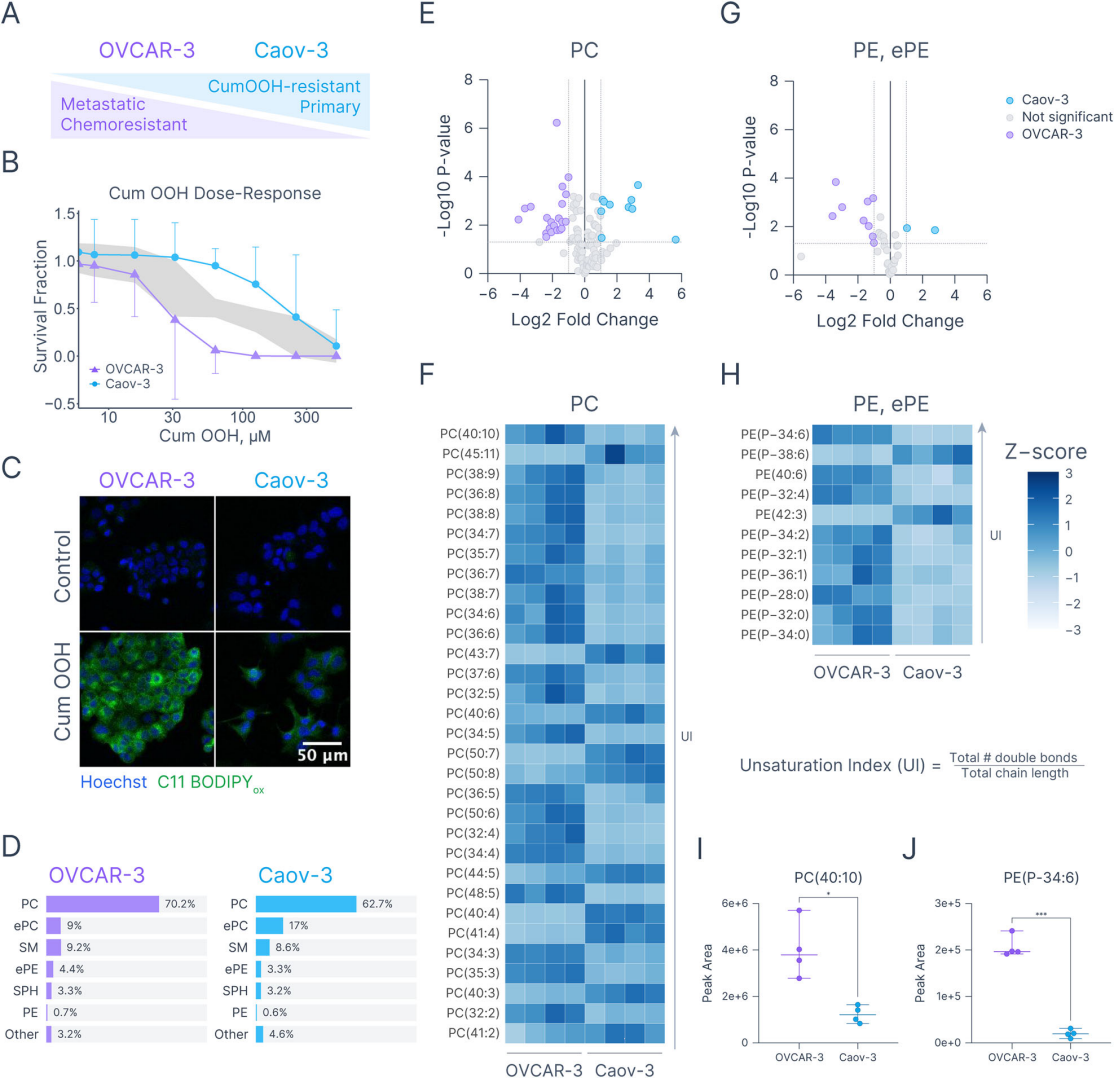
Baseline differences in lipid peroxidation sensitivity and lipid composition in OVCAR-3 and Caov-3 cells. **A** Metastatic and relatively chemoresistant OVCAR-3 cells are more sensitive to Cum OOH-induced lipid peroxidation than primary and chemosensitive Caov-3 cells. **B** Dose-response curves showing differential sensitivity to Cum OOH between OVCAR-3 and Caov-3 cells. **C** Oxidized C11 BODIPY fluorescence in OVCAR-3 and Caov-3 cells treated with 50 μM Cum OOH for 1.5 h. Blue – Hoechst fluorescence, green – oxidized C11 BODIPY fluorescence. **D** Relative abundances of select lipid classes in OVCAR-3 and Caov-3 cells. PC phosphatidylcholines, SM sphingomyelins, ePC ether-linked phosphatidylcholines, ePE ether-linked phosphatidylethanolamines, SPH sphingosines, PE phosphatidylethanolamines. Volcano plot (**E**) and a heatmap (**F**) depicting differentially expressed PCs between OVCAR-3 and Caov-3 cells. Volcano plot (**G**) and a heatmap (**H**) depicting differentially expressed PEs between OVCAR-3 and Caov-3 cells. Arrows indicate an increase in unsaturation index calculated as the total carbon chain length divided by the total number of double bonds. Peak areas of PC(40:10) (**I**) and PE(P-34:6) (**J**) as representative polyunsaturated phospholipids. Statistics: **B** Each point represents the mean of three biological replicates, each conducted in duplicate. Gray area denotes the null zone with non-overlapping 95% confidence intervals. **E**, **G** Each data point represents the mean of four biological replicates. Differentially expressed lipids are defined as those with absolute log2 fold change > 1 and *p*-value < 0.05 (two-tailed unpaired t-test). **F**, **H** Heatmaps represent z scores of lipid species with absolute log2 fold change > 1 and *p*-value < 0.05 (two-tailed unpaired t-test), four biological replicates. **I**, **J** Each dot represents a biological replicate; error bars represent the range. Statistical significance was determined using two-tailed unpaired t-test. *p ≤ 0.05, ***p ≤ 0.001. Heatmaps and the null zone dose-response plot were created in R (version 4.4.1 2024-06-14), volcanos and spread plots were created with Graphmatik (version 0.3.2, 2025).

To conduct a mechanistic comparison of pharmacological ferroptosis and PDT-induced cell death, two HGSOC cell lines were selected – OVCAR-3 and Caov-3. These cell lines represent primary (Caov-3) and metastatic (OVCAR-3) HGSOC with high and low sensitivity to standard-of-care chemotherapy, respectively (Fig. 1A, S1) [59, 60]. Treatment with escalating doses of cumene hydroperoxide (Cum OOH), a well-characterized lipid oxidant [61], revealed that these cell lines exhibit differential sensitivity to lipid oxidation (Fig. 1B, S2 Table S1). Treatment with 31.25 μM Cum OOH for 72 h resulted in survival fractions of 0.38 ± 0.19 in OVCAR-3 and 1.04 ± 0.08 in Caov-3 cells. To investigate Cum OOH-induced generation of lipid radicals, cells were pre-stained with C11 BODIPY, a well-established fluorescence sensor. Oxidation of the polyunsaturated butadienyl residue within the C11 BODIPY molecule leads to a shift of the fluorescence emission peak from ~590 nm to ~510 nm, enabling ratiometric measurements of lipid RMS flux [62, 63]. Treatment with 50 μM Cum OOH for 1.5 h led to a robust increase in oxidized C11 BODIPY in OVCAR-3, but not Caov-3 cells, as demonstrated by confocal microscopy (Fig. 1C) [62]. It was hypothesized that differences in Cum OOH sensitivity may, at least in part, arise from variations in the underlying lipid composition, particularly in the amount of unsaturated membrane-forming phospholipids. In these lipids, hydrogen abstraction from allylic or bis-allylic positions can lead to the formation of a lipid radical with the subsequent oxygenation and formation of peroxyl radicals [64]. To test this hypothesis, lipidomic profiling was conducted, resulting in the identification of 594 unique lipid species, out of which 241 were differentially expressed (Fig. 1D, S3 & Table S2). Subsequent analysis of phosphatidylcholines (PCs), the most abundant phospholipid class, and phosphatidylethanolamines (PEs, including plasmalogens - pPE), a class of phospholipids strongly implicated in ferroptosis [20], identified 31 PCs and 11 PEs with absolute log2 fold change > 1 and *p*-value < 0.05. Interestingly, 18 out of 31 PC species overrepresented in OVCAR-3 cells contained more than 4 double bonds (Fig. 1E–H). For example, PC(40:10), a differentially expressed PC with the highest ratio between the total number of double bonds and carbon chain length (unsaturation index), was significantly overrepresented in OVCAR-3 cells (peak area of 4.02 × 10^6^ in OVCAR-3 versus 1.23 × 10^6^ in Caov-3, *p*-value = 0.016, Fig. 1I). Similarly, differentially expressed PE with the highest unsaturation index PE(P-34:6) was present at a higher concentration in OVCAR-3 cells (peak area of 2.07 × 10^5^ in OVCAR-3 versus 1.99 × 10^4^ in Caov-3 cells, *p*-value = 0.0002, Fig. 1J).

### OVCAR-3 and Caov-3 cells exhibit distinct sensitivities to pharmacological ferroptosis

**Fig. 2:**
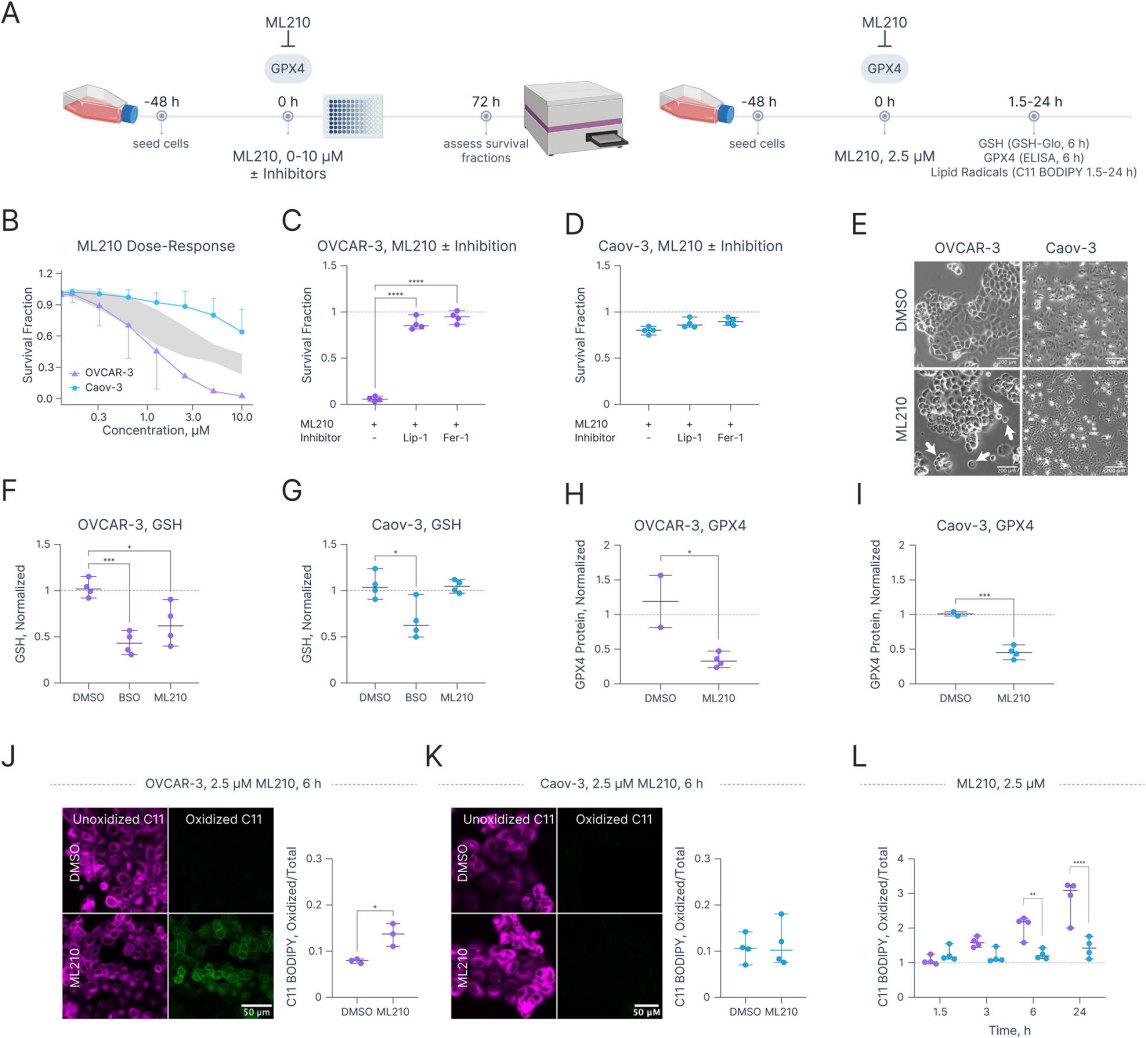
OVCAR-3 and Caov-3 cells exhibit differential sensitivity to ML210-induced ferroptosis and lipid peroxidation. **A** Experimental workflow schematics depicting ML210 dose responses +/− inhibition as well as the effects of treatment with 2.5 μM ML210 for 6 h on intracellular GSH levels, GPX4 protein concentration and lipid radical generation. Created in BioRender. Overchuk, M. (2025) https://BioRender.com/6u41lj7. **B** ML210 dose-response curves in OVCAR-3 and Caov-3 cells. Survival fraction in (**C**) OVCAR-3 and (**D**) Caov-3 cells treated with 5 μM ML210 with or without 2.5 μM Lip-1 or Fer-1 for 72 h. **E** Widefield microscopy images of OVCAR-3 and Caov-3 cells treated with 2.5 μM ML210 for 6 h. Scale bar = 200 μm. Relative change in intracellular GSH concentrations in (**F**) OVCAR-3 and (**G**) Caov-3 cells treated with 500 μM BSO or 2.5 μM ML210 for 6 h. Relative change in GPX4 protein contents in (**H**) OVCAR-3 and (**I**) Caov-3 cells treated with 2.5 μM ML210 for 6 h. Confocal microscopy images of unoxidized and oxidized C11 BODIPY fluorescence and relative change in oxidized/total C11 BODIPY fluorescence ratio in (**J**) OVCAR-3 and (**K**) Caov-3 cells. **L** Time-dependent changes in relative oxidized/total C11 BODIPY fluorescence in OVCAR-3 and Caov-3 cells measured in a plate reader. Statistics: **B** Each dot represents the mean of at least three biological replicates, each performed in duplicate. Error bars represent 95% confidence intervals, gray area indicate the “null zone,” where 95% confidence intervals do not overlap. **C**, **D**, **F**, **G**, **H**, **I**, **L** Each dot represents the mean of a biological replicate conducted in duplicate; error bars represent the range. **J**, **K** Each dot represents a biological replicate mean, calculated from ≥10 fields of view. Statistical significance was determined using two-tailed unpaired t-test (**H**–**K**), one-way ANOVA with Dunnett correction for multiple comparisons (**C**, **D**, **F**, **G**) or two-way ANOVA with Šidák correction for multiple comparisons (**L**). *p ≤ 0.05, **p ≤ 0.01, ***p ≤ 0.001, ****p ≤ 0.0001. The null zone dose-response plot was created in R (version 4.4.1 2024-06-14), spread plots were created in Graphmatik (version 0.3.2, 2025).

Given the observed differences in sensitivity to Cum OOH and phospholipid unsaturation between OVCAR-3 and Caov-3 cells, it was hypothesized that these cell lines exhibit distinct ferroptosis sensitivity profiles. Cells were treated with escalating concentrations of a canonical ferroptosis inducer ML210 **(**Fig. 2A) [8, 9]. Upon entering cells, ML210 covalently binds the catalytic selenocysteine 46 residue of GPX4. Consequently, GPX4 inactivation enables the propagation of lipid oxidative damage leading to ferroptosis. OVCAR-3 cells demonstrated a robust response, wherein treatment with 10 μM ML210 resulted in an almost complete elimination of viable cells. Caov-3 cells, on the other hand, were significantly more resistant. The same treatment with 10 μM ML210 resulted in a survival fraction of 0.64 ± 0.09 (Fig. 2B & S4, Table S3). These data agree with those of Cum OOH sensitivity and lipidomic analysis indicating that OVCAR-3 cells were more sensitive to lipid peroxidation compared to Caov-3 cells. OVCAR-3 cells could be successfully rescued from ML210-induced ferroptosis upon the addition of lipophilic radical-trapping antioxidants (liproxstatin-1, Lip-1 and ferrostatin -1, Fer-1, Fig. S5) [65]. Treatment with ML210 at 5 μM for 72 h decreased the survival fraction to 0.06 ± 0.01, while co-incubation with Lip-1 and Fer-1 restored survival fractions to 0.87 ± 0.03 and 0.94 ± 0.03, respectively (Fig. 2C). Although no changes in Caov-3 survival fractions were observed at the equivalent conditions (Fig. 2D, E), Lip-1 and Fer-1 were protective at lower cell densities wherein ML210 exhibited cytotoxic effects (Fig. S6). Next, the effects of ML210 treatment were investigated in relation to the two key ferroptosis defense mechanisms: intracellular GSH concentration and GPX4 expression. Although treatment with L-buthionine-sulphoximine (BSO), an inhibitor of gamma-glutamylcysteine synthetase (Fig. S7, S8), significantly decreased relative GSH content in both cell lines, treatment with ML210 (2.5 μM, 6 h) depleted GSH only in ferroptosis-sensitive OVCAR-3 (0.64 ± 0.11, *p*-value = 0.01), but not ferroptosis-resistant Caov-3 (1.05 ± 0.03, *p*-value = 0.99) cells (Fig. 2F, G & S9). However, relative GPX4 protein content was significantly lower in both ML210-treated OVCAR-3 (0.34 ± 0.05, *p*-value = 0.02) and Caov-3 cells (0.45 ± 0.05, *p*-value = 0.001) compared to vehicle-treated cells (Fig. 2H, I).

Next, the C11 BODIPY fluorescent sensor was used to measure the accumulation of lipid radicals following ML210 treatment. Confocal microscopy of C11 BODIPY-stained OVCAR-3 cells demonstrated increased oxidized C11 BODIPY fluorescence in ML210-treated OVCAR-3 (Figs. 2J, S10, S11), but not Caov-3 (Fig. 2K) cells. This trend was confirmed with a plate reader assay (Fig. 2L), wherein the oxidized/total C11 BODIPY fluorescence ratio steadily increased at 1.5, 3, 6, and 24 h in OVCAR-3, but not Caov-3 cells. To summarize, OVCAR-3 and Caov-3 cells show significantly different sensitivities to the canonical ferroptosis inducer ML210, reflecting differences in phospholipid unsaturation and their respective sensitivities to chemical lipid oxidation by Cum OOH.

### PDT induces lipid radicals in both ferroptosis-sensitive and -resistant cells

**Fig. 3:**
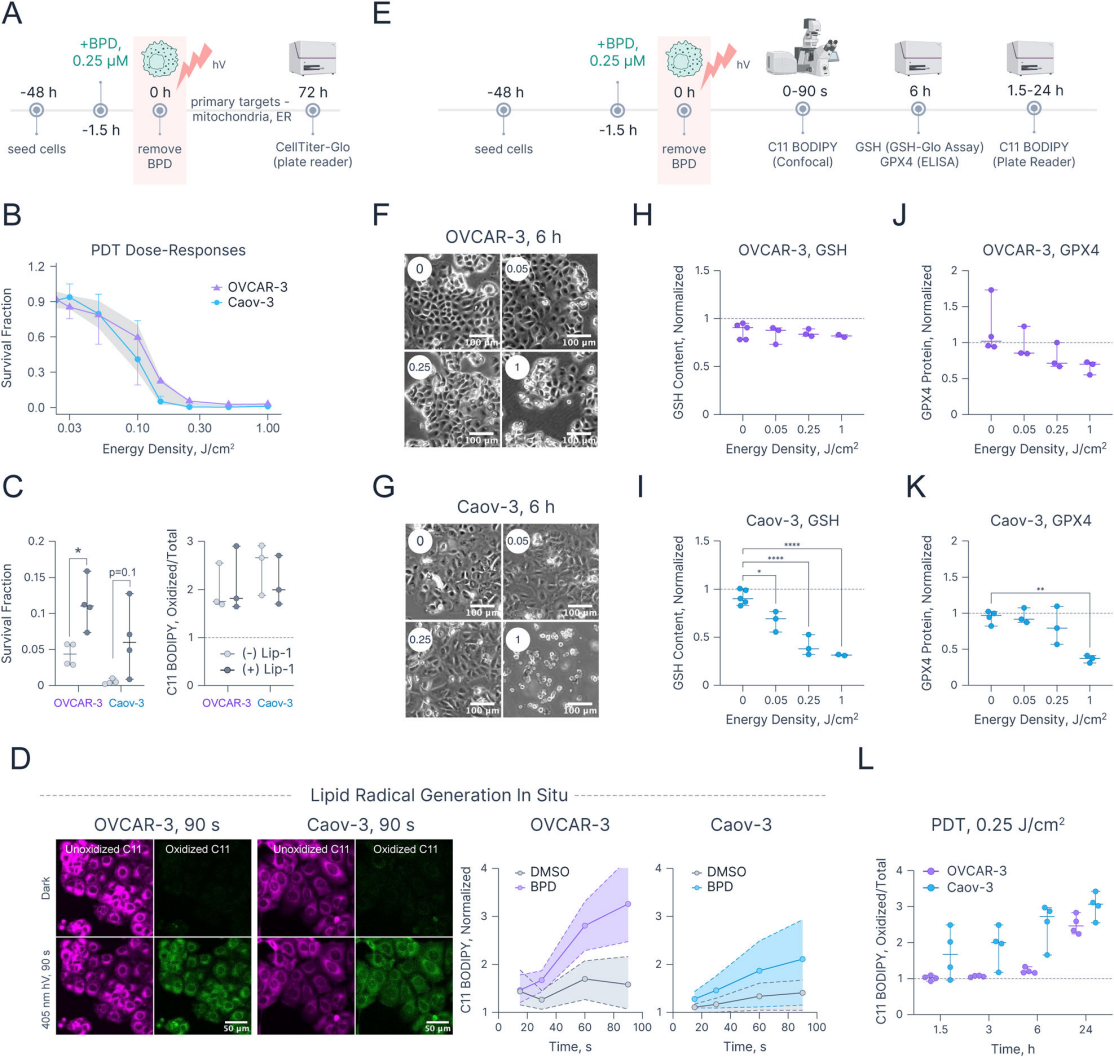
PDT-induced lipid radical generation and antioxidant response. **A** Experimental workflow for PDT in OVCAR-3 and Caov-3 cells. Created in BioRender. Overchuk, M. (2025) https://BioRender.com/sgtz48i. **B** PDT dose-response curves in OVCAR-3 and Caov-3 cells. **C** Survival fractions and lipid radical generation induced by PDT at 0.25 J/cm^2^ in OVCAR-3 and Caov-3 cells with and without 10 μM Lip-1. **D** Confocal microscopy images of unoxidized and oxidized C11 BODIPY fluorescence in BPD-treated OVCAR-3 and Caov-3 cells after 90 s of 405 nm light exposure (scale bar = 50 μm) as well as time-dependent changes in relative oxidized/total C11 BODIPY fluorescence in OVCAR-3 and Caov-3 cells based on confocal images. **E** Experimental workflow for C11 BODIPY, GSH, and GPX4 assays post-PDT. Created in BioRender. Overchuk, M. (2025) https://BioRender.com/sgtz48i. Widefield microscopy images of (**F**) OVCAR-3 and (**G**) Caov-3 cells 6 h after being treated with PDT at 0.05, 0.25, and 1 J/cm^2^. Scale bar = 100 μm. Relative change in intracellular concentration of GSH in (**H**) OVCAR-3 and (**I**) Caov-3 cells. Relative GPX4 protein content in (**J**) OVCAR-3 and (**K**) Caov-3 cells 6 h post-PDT treatment. **L** Time-dependent changes in relative oxidized/total C11 BODIPY fluorescence in OVCAR-3 and Caov-3 cells measured in a plate reader. Statistics: **B** Each dot represents the mean of at least three biological replicates, each conducted in duplicate. Error bars represent 95% confidence intervals, gray area represents the “null zone,” where 95% confidence intervals do not overlap. **C**, **H**, **I**, **J**, **K**, **L** Each dot represents the mean of a biological replicate, each conducted in duplicate; error bars represent the range. **D** Each dot represents the mean of 3 biological replicates, each containing at least five fields of view. Shaded areas represent the range. Statistical significance was determined using one-way ANOVA with Dunnett correction for multiple comparisons for multiple comparisons (**H**–**K**) or two-way ANOVA with Šidák correction for multiple comparisons (**C**, **L**). *p ≤ 0.05, **p ≤ 0.01, ***p ≤ 0.001, ****p ≤ 0.0001. The null zone dose-response plot was created in R (version 4.4.1 2024-06-14), spread and line charts were created in Graphmatik (version 0.3.2, 2025).

Next, the ability of PDT using BPD to initiate lipid peroxidation and ferroptosis-like cell death was investigated in OVCAR-3 and Caov-3 cell lines. Cells were treated with 0.25 μM BPD for 90 min and exposed to escalating energy densities (0–1 J/cm^2^) of 690 nm light (Fig. 3A). Prior studies, including our own, have shown that this PDT protocol enables BPD internalization and its accumulation within mitochondrial and ER membranes [49, 66]. Unlike pharmacological ferroptosis, PDT demonstrated comparable potency in both cell lines resulting in similar decreases in survival fractions at 72 h (Fig. 3B). To investigate the potential contribution of lipid peroxidation to PDT-induced cytotoxicity, cells were co-incubated with BPD and 10 μM of Lip-1 prior to light exposure (Figs. 3C, S12). Co-incubation with Lip-1 significantly rescued OVCAR-3 cells from PDT-induced cytotoxicity at 0.25 J/cm^2^, increasing survival fractions of 0.04 ± 0.01 to 0.11 ± 0.02 (*p*-value = 0.02, Figs. 3C, S13). In Caov-3 cells, a similar trend toward increased survival fraction was observed (0.005 ± 0.002 versus 0.05 ± 0.02), though it did not reach statistical significance (*p*-value = 0.1). Notably, despite the partial protection against cell death in both cell lines, Lip-1 did not reduce lipid radical generation as measured by the C11 BODIPY assay at 24 h post-PDT. This suggests that Lip-1, at the tested concentration, may be insufficient to suppress photochemically-induced RMS formation.

To investigate how photosensitizer localization influences the photochemical generation of lipid radicals, we developed an alternative PDT regimen with a short drug–light interval (SDLI-PDT). In this protocol, BPD was added to cells immediately before light exposure. Because the BPD incubation time was brief (<2 min), PDT-induced RMS damage likely occurred at or near the cell surface, primarily affecting the plasma membrane rather than the subcellular organelles typically targeted after longer incubation (90 min) [51]. SDLI-PDT with 2.5 μM BPD produced dose–response profiles comparable to those obtained with the conventional PDT regimen in both cell lines (Fig. S14). We next examined whether pre-incubation with Lip-1 could protect cells from SDLI-PDT. Similar to the conventional protocol, Lip-1 conferred significant protection in OVCAR-3 cells and showed a trend toward increased survival in Caov-3 cells (Fig. S15). Notably, unlike the conventional regimen, Lip-1 significantly reduced lipid radical generation in OVCAR-3 cells subjected to SDLI-PDT at 0.25 J/cm^2^. These findings indicate that lipid radical generation plays a role in PDT-induced cytotoxicity in both mitochondrial/ER-targeting and SDLI-PDT regimens.

To further investigate the ability of PDT to generate lipid radicals in situ, C11 BODIPY ratiometric fluorescence was examined using confocal microscopy. To capture lipid radical generation immediately post light irradiation, cells were pre-stained with BPD and 10 μM C11 BODIPY, and BPD was excited at 405 nm (Soret peak) using a confocal laser for 15–90 s. A time-dependent increase in oxidized/total C11 BODIPY fluorescence ratio was observed in both OVCAR-3 and Caov-3 cells (Fig. 3D), although it was more evident in OVCAR-3 cells. Next, the effects of PDT at 0.05, 0.25, and 1 J/cm^2^ on GSH content, GPX4 expression and lipid radical generation were investigated 6 h post PDT (Fig. 3E). The same PDT doses led to comparable decreases in survival fractions at 6 h post treatment in both cell lines (Fig. S16), however, Caov-3 cells showed more extensive cell detachment at 1 J/cm^2^ compared to OVCAR-3 cells (Fig. 3F, G). Interestingly, this coincided with dose-dependent changes in intracellular GSH concentration and GPX4 expression in this cell line (Fig. 3H–K). OVCAR-3 cells showed no significant change in GSH content and only a minor decrease in GPX4 protein concentration post-PDT. Finally, time-dependent generation of lipid radicals was examined at 1.5, 3, 6, and 24 h post-PDT using a plate reader-based C11 BODIPY assay (Fig. 3L). Interestingly, both cell lines exhibited a marked increase in the ratio of oxidized to total C11-BODIPY fluorescence at 24 h post-treatment. To summarize, our data indicate that PDT is capable of inducing lipid RMS generation in both cell lines, although these effects are less prominent in ferroptosis-resistant Caov-3 cells.

### PDT-induced cytotoxicity and lipid radical generation is independent of intracellular iron

**Fig. 4:**
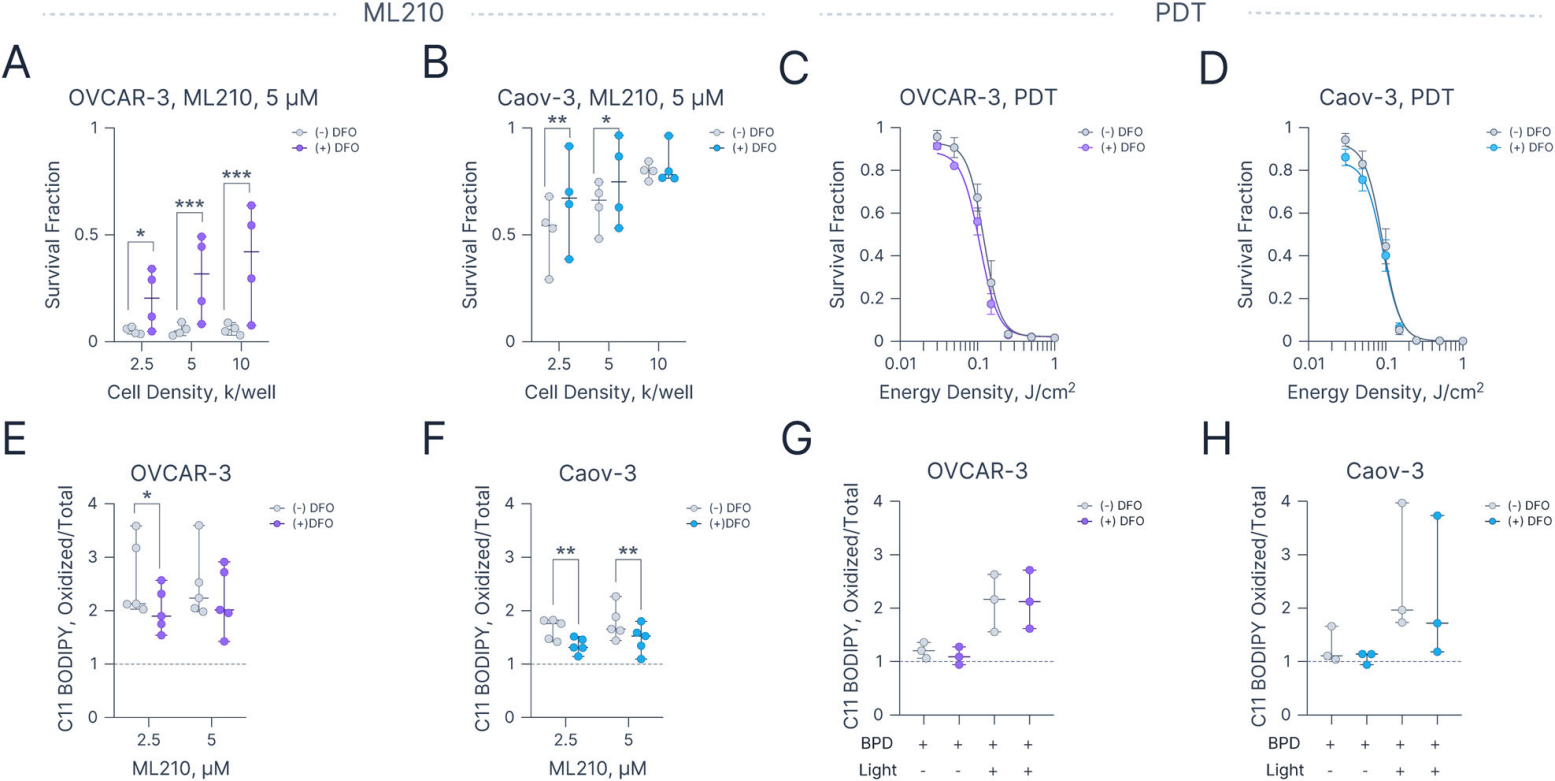
Effects of iron chelation on ML210- and PDT-induced cytotoxicity and lipid peroxidation. Survival fractions of (**A**) OVCAR-3 and (**B**) Caov-3 cells seeded at 0.25, 0.5, and 1 × 10^4^ cells/well and treated with 5 μM ML210 ± 1.25 μM DFO for 72 h. Survival fractions of (**C**) OVCAR-3 and (**D**) Caov-3 cells following escalating PDT doses with or without 25 μM DFO pre-treatment (1.5 h). Lipid radical generation in (**E**) OVCAR-3 and (**F**) Caov-3 cells treated with 2.5 or 5 μM ML210 ± 1.25 μM DFO for 24 h, measured as the ratio of oxidized to total C11 BODIPY fluorescence, normalized to no treatment control. Lipid radical generation in (**G**) OVCAR-3 and (**H**) Caov-3 cells treated with 0.25 J/cm² PDT ± 25 μM DFO, measured as the ratio of oxidized to total C11 BODIPY fluorescence, normalized to no treatment control. Statistics: **A**, **B**, **E**, **F**, **G**, **H** Each dot represents the mean of a biological replicate each conducted in duplicate; error bars represent the range. **C**, **D** Each dot represents the mean of at least three biological replicates each conducted in duplicate, error bars represent the standard error of the mean. Lines represent a four-parameter nonlinear regression fit (R-squared > 0.99). Statistical significance was determined using two-way ANOVA with Šídák’s correction for multiple comparisons. *p ≤ 0.05, **p ≤ 0.01, ***p ≤ 0.001. All plots were created in Graphmatik (version 0.3.2, 2025).

As the name suggests, canonical ferroptosis is characterized by its dependency on intracellular iron levels [8]. To explore the role of iron in both ML210- and PDT-induced cell death, we utilized clinical iron chelator deferoxamine (DFO, Fig. 4). We first determined sub-cytotoxic DFO concentrations at two different exposure durations: 72 h, mimicking ML210 experiments, and 1.5 h, mimicking BPD exposure for PDT experiments (Fig. S17). Interestingly, OVCAR-3 cells were significantly more sensitive to iron chelation than Caov-3 cells, requiring the use of a low DFO concentration (1.25 μM) to avoid compromising cell viability at 72 h (Fig. S17A). Despite this low concentration, DFO significantly rescued both OVCAR-3 and Caov-3 cells from ML210-induced cytotoxicity (Fig. 4A, B). For instance, treatment with 5 μM ML210 reduced the survival fraction of OVCAR-3 cells (1 × 10^4^ cells per well) to 0.05 ± 0.01, whereas co-incubation with DFO significantly increased survival to 0.38 ± 0.12 (*p*-value = 0.0002) under identical conditions. Since both cell lines tolerated a 1.5 h exposure to 25 μM DFO, this concentration was used in subsequent PDT experiments (Fig. S17B, C). In contrast to ML210 experiments, exposure to 25 μM DFO for 1.5 h had no protective effect against PDT-induced cell death in either cell line (Fig. 4C, D), nor did it rescue cells subjected to SDLI-PDT (Fig. S18). We next assessed the effect of iron chelation on ML210- and PDT-induced lipid radical generation. In OVCAR-3 cells, DFO significantly reduced lipid radical levels at 2.5 μM ML210 but not at 5 μM, possibly due to the high degree of lipid unsaturation in this cell line, which favors lipid radical propagation (Fig. 4E, F). In Caov-3 cells, DFO significantly decreased lipid radical formation at both ML210 concentrations. In contrast, DFO had no impact on PDT-induced lipid radical generation in either cell line, further supporting the iron-independent nature of PDT-induced lipid oxidation (Fig. 4G, H).

### PDT induces dose-dependent phospholipid oxidation resembling ML210-induced ferroptosis

**Fig. 5:**
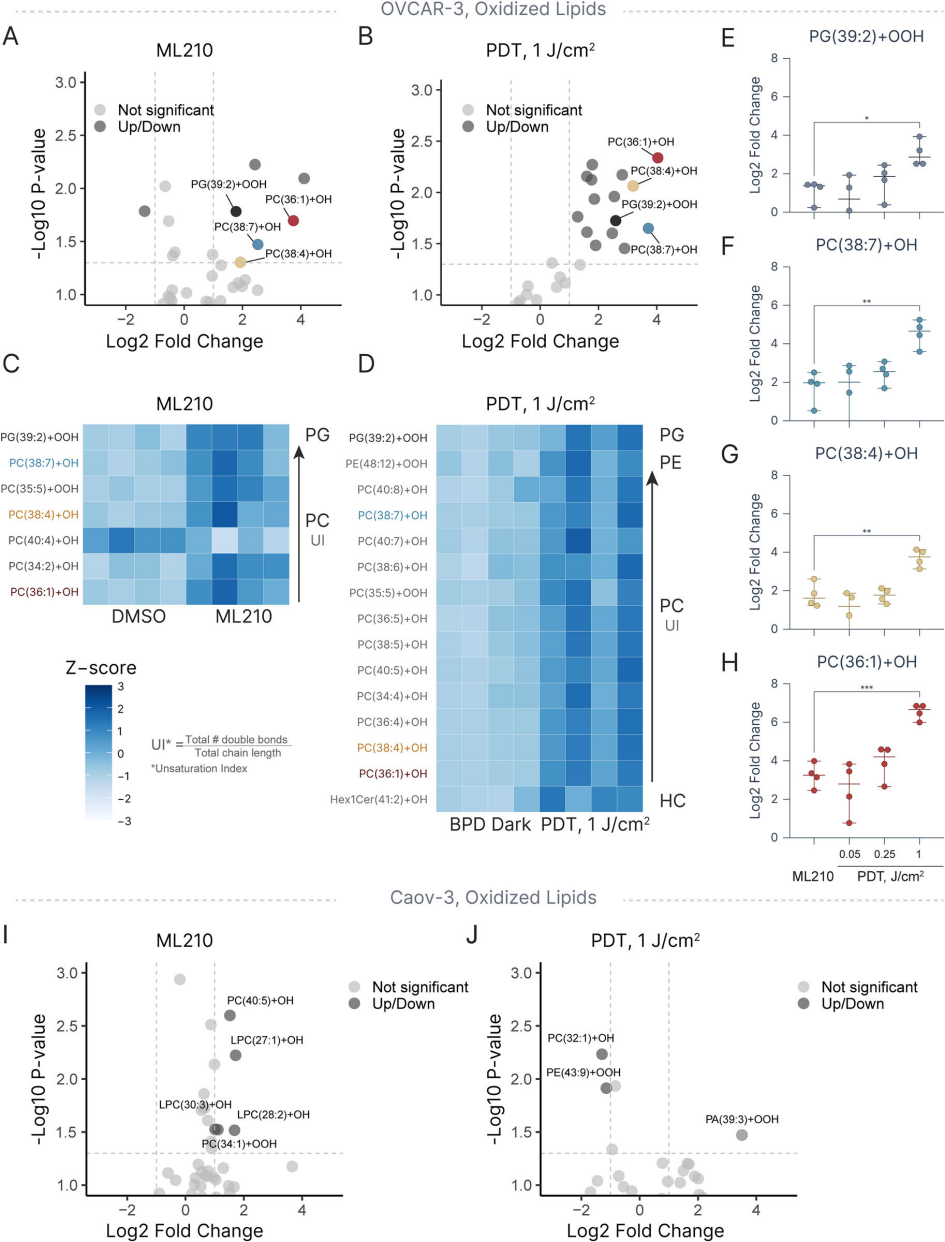
Oxidized lipids induced by ML210 and PDT. Volcano plots demonstrating differentially expressed lipid hydroxides and hydroperoxides in OVCAR-3 cells treated with (**A**) ML210 and (**B**) PDT at 1 J/cm^2^. Heatmaps depicting z scores of differentially expressed oxidized lipids in (**C**) ML210- and (**D**) PDT-treated OVCAR-3 cells. **E**, **F**, **G**, **H** Plots showing log2 fold changes of the four oxidized lipid species significantly upregulated by both ML210 and PDT. Volcano plots demonstrating differentially expressed lipid hydroxides and hydroperoxides in Caov-3 cells treated with (**I**) ML210 and (**J**) PDT at 1 J/cm^2^. Statistics: **A**, **B**, **I**, **J** Each data point represents the mean of four biological replicates. Differentially expressed lipids are defined as those with absolute log2 fold change > 1 and *p*-value < 0.05 (two-tailed unpaired t-test). **C**, **D** Differentially expressed species are defined as those with absolute log2 fold change > 1 and *p*-value < 0.05 (two-tailed unpaired t-test). **E**–**H** Each dot represents the mean of a biological replicate, each conducted in duplicate; error bars represent the range. Statistical significance was determined using one-way ANOVA with Dunnett correction for multiple comparisons. *p ≤ 0.05, **p ≤ 0.01, ***p ≤ 0.001. Heatmaps and volcanos were created in R (version 4.4.1 2024-06-14), spread plots were created with Graphmatik (version 0.3.2, 2025).

Next, the effects of ML210 and three PDT doses on the relative abundance of hydroxy- and hydroperoxy- lipid derivatives were interrogated. A total of 292 -OH and -OOH-containing lipids were identified through LipidSearch. Manual chromatogram review narrowed down this list to 79 oxidized lipids with well-resolved chromatogram peaks. In OVCAR-3 cells, treatment with ML210 resulted in a significant upregulation (absolute log2 fold change > 1 and *p*-value < 0.05) of 6 oxidized lipids, including PC(34:2), PC(35:5), PC(36:1), PC(38:4), PC(38:7), and PG(39:2) **(**Fig. 5A**)**. Following PDT at the highest dose (1 J/cm^2^), a significant upregulation of 15 oxidized lipids was detected in the same cell line, demonstrating that PDT is capable of targeting a broader set of lipid species compared to ML210 (Fig. 5B & S19, S20). Interestingly, a drill-down analysis revealed a significant overlap between oxidized lipids upregulated by ML210 and PDT (Fig. 5C, D). Specifically, PC(36:1) + H + OH, PC(38:4) + H + OH, PC(38:7) + H + OH, and PG(39:2) + H + OH were upregulated by both ML210 and PDT, wherein treatment with PDT at 0.05, 0.25, and 1 J/cm^2^ resulted in a progressive increase in relative abundances of these species (Fig. 5E–H). It is important to note that in all cases, the subtherapeutic PDT dose of 0.05 J/cm^2^ (<IC10) led to similar amounts of these species compared to ML210 at 2.5 μM (IC50), and PDT at 1 J/cm^2^ resulted in a significantly higher oxidized lipid content compared to ML210. In ferroptosis-resistant Caov-3 cells, ML210 treatment upregulated 5 oxidized lipids, whereas PDT upregulated only 1 (Fig. 5I, J). The lack of detectable oxidized lipids in PDT-treated Caov-3 cells may be explained by two possibilities: (1) a lower overall abundance of oxidized lipids formed due to a higher degree of phospholipid saturation, or (2) differing kinetics of cell death between the cell lines, suggesting that any oxidized phospholipids in Caov-3 cells may have been degraded prior to our 6-hour measurement. Overall, these results underscore that the ability of PDT to induce lipid peroxidation is highly dependent on underlying cell phenotypes and lipid composition.

### PDT upregulates ceramides in both ferroptosis-sensitive and -resistant cells

**Fig. 6:**
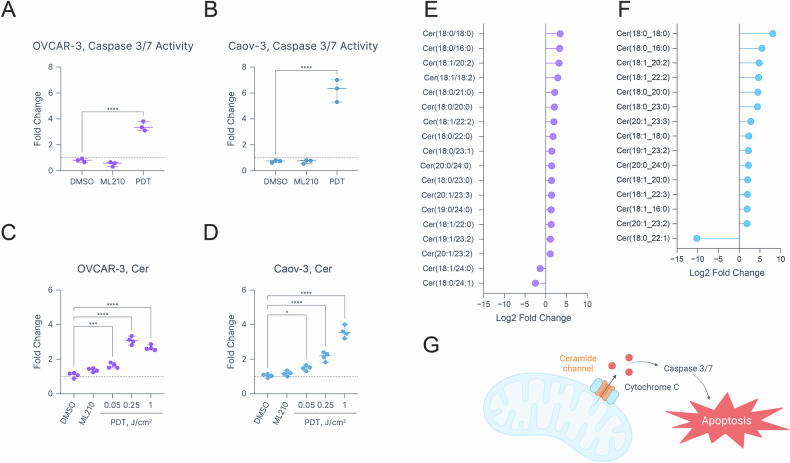
PDT-induced apoptosis in OVCAR-3 and Caov-3 cells. Upregulation of cleaved caspase 3/7 in PDT- and ML210-treated (**A**) OVCAR-3 and (**B**) Caov-3 cells. Relative Cer content in (**C**) OVCAR-3 and (**D**) Caov-3 cells treated with 2.5 μM ML210 and PDT at 0.05, 0.25, and 1 J/cm^2^. Significantly up- or down-regulated Cer species affected by PDT treatment (1 J/cm^2^) in (**E**) OVCAR-3 and (**F**) Caov-3 cells. **G** Schematic representation of Cer involvement in apoptosis induction. Created in BioRender. Overchuk, M. (2025) https://BioRender.com/u9cv1qh. Statistics: **A**–**D** Each dot represents the mean of a biological replicate each conducted in duplicate; error bars represent the range. One-way ANOVA with Dunnett correction for multiple comparisons; **p ≤ 0.01, ***p ≤ 0.001, ****p ≤ 0.0001. **E**, **F** Points represent medians of four biological replicates. Only significant species (absolute log2 fold changes > 1, *p*-value < 0.05 determined with unpaired t-test) are included. All plots were created in Graphmatik (version 0.3.2, 2025).

Despite the differential sensitivity to lipid oxidation in OVCAR-3 and Caov-3 cells, PDT exhibited comparable efficacy. Given this observation, we hypothesized that PDT-induced cell killing involves a combination of lipid peroxidation and apoptosis. We hypothesized that Caov-3 cells, being relatively chemosensitive and ferroptosis-resistant, would be more prone to PDT-induced apoptosis than the relatively chemoresistant and ferroptosis-sensitive OVCAR-3 cells. To quantify apoptosis induction, caspase 3/7 activation was measured via a luminescence assay in ML210- and PDT (0.25 J/cm^2^)-treated cells at 6 h (Fig. 6A, B). While PDT treatment significantly upregulated active caspase 3/7 in both cell lines compared to DMSO, the relative increase was notably higher in Caov-3 cells (6.22 ± 0.49, *p*-value < 0.0001) than in OVCAR-3 cells (3.42 ± 0.2, *p*-value < 0.0001). Next, the effects of ML210 and PDT on global lipid composition were investigated. Although a multitude of lipid classes were affected by PDT, Cer were consistently upregulated in a dose-dependent manner in both cell lines (Fig. 6C–F, S21, S22, S23 & S24). Specifically, PDT at 1 J/cm^2^ increased Cer content 2.66 ± 0.07 times in OVCAR-3 cells and 3.57 ± 0.17 times in Caov-3 cells compared to controls. These findings are consistent with the literature indicating that PDT promotes de novo Cer synthesis, a critical step in mitochondrial membrane permeabilization and apoptosis (Fig. 6G) [52, 53]. Additionally, the relatively greater upregulation of apoptosis-inducing Cer in Caov-3 cells aligns with the comparatively higher activation of caspase 3/7 observed in these cells. ML210, conversely, had minimal effects on both caspase 3/7 and Cer content. Aside from changes in Cer content, PDT exhibited dose- and cell line-dependent effects on hexosylceramides, phosphatidylinositols, diglycerides, and cardiolipins, indicating lipidome-wide rearrangements.

### PDT-induced lipidomic changes in an OVCAR-3 xenograft mouse model

**Fig. 7:**
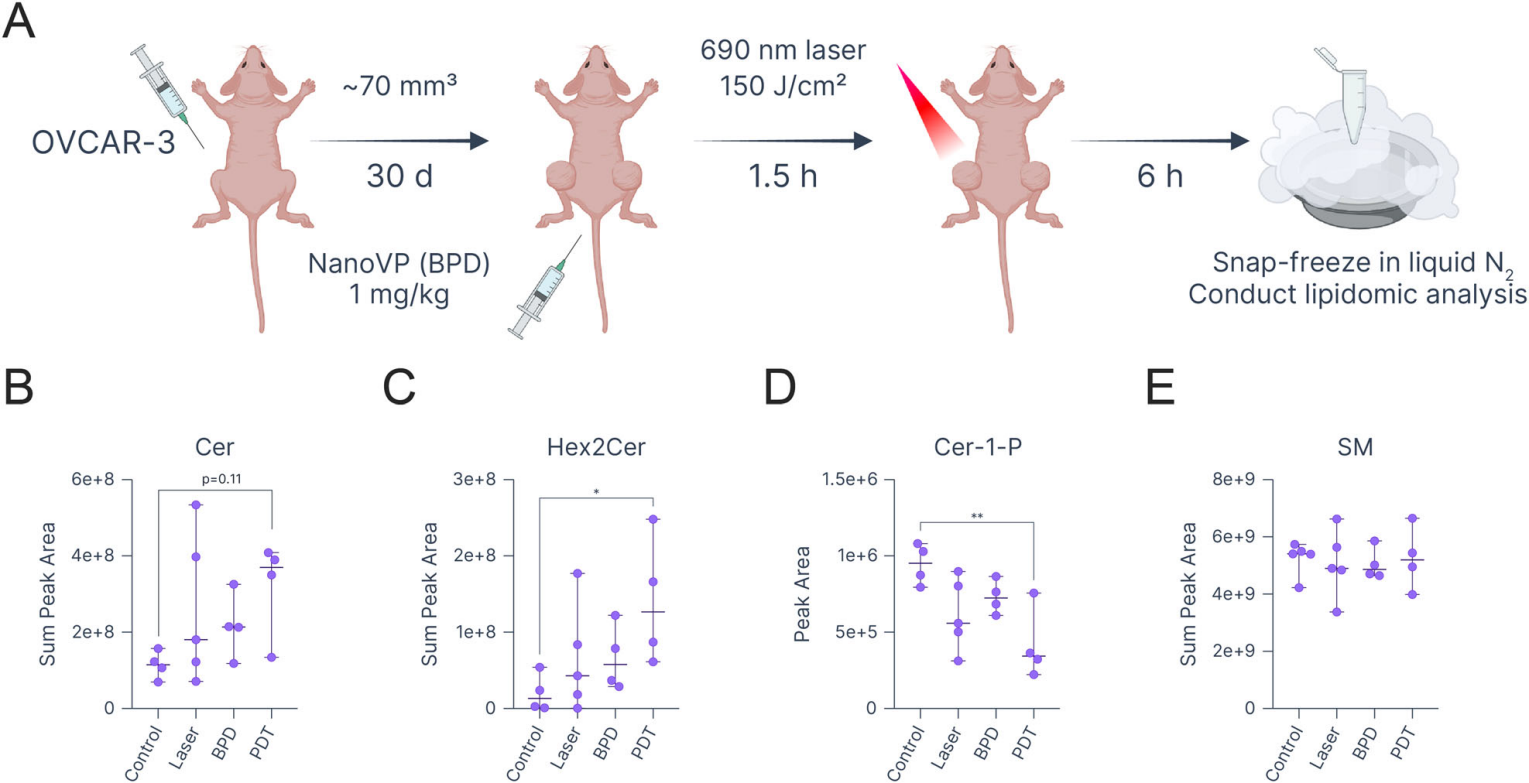
PDT-induced lipidomic changes in a subcutaneous OVCAR-3 mouse xenograft model. **A** Experimental workflow schematic. Created in BioRender. Overchuk, M. (2025) https://BioRender.com/uv606us. Sum peak areas of (**B**) Cer and (**C**) Hex2Cer. Peak areas of (**D**) Cer-1-P and sum peak areas of (**E**) SM in control, laser-, BPD- and PDT-treated tumor tissues. Statistics: **B**–**E** Each dot represents a biological replicate, error bars represent the range. One-way ANOVA with Dunnett correction for multiple comparisons; *p ≤ 0.05, **p ≤ 0.01. All plots were created with Graphmatik (version 0.3.2, 2025).

To investigate the effects of PDT on lipid composition in vivo, athymic J:NU mice bearing bilateral OVCAR-3 tumors were injected with either saline or a nanoformulated BPD (NanoVP) at 1 mg/mL or equal volume of saline [55, 56]. After 1.5 h, one tumor per mouse was exposed to 690 nm light at 150 J/cm^2^ resulting in four types of tumor samples: control, laser-only, BPD-only, and PDT (Fig. 7A). Lipidomic analysis revealed a trend towards increased Cer concentrations in PDT-treated tumors compared to the control tumors (control: 1.1 × 10^8^ ± 1.8 × 10^7^, PDT: 3.2 × 10^8^ ± 6.33 × 10^7^, *p*-value = 0.11, Fig. 7B). This trend was even more pronounced for Cer disaccharides (Hex2Cer), less active forms of Cer formed by the sequential addition of two hexose sugars to a Cer molecule (control: 2.02 × 10^7^ ± 1.23 × 10^7^ versus PDT: 1.4 × 10^8^ ± 4.2 × 10^7^, *p*-value = 0.039, Fig. 7C). Interestingly, the increase in Cer was accompanied by a significant reduction in Cer-1-P, a Cer metabolite known for its potent anti-apoptotic and pro-survival roles (control: 9.44 × 10^5^ ± 6.65 × 10^4^ versus PDT: 4.16 × 10^5^ ± 1.17 × 10^5^, *p*-value = 0.01, Fig. 7D) [54]. No changes in SM, a common Cer precursor, were observed, likely indicating that the observed Cer increase stems from de novo synthesis rather than sphingomyelins (SM) breakdown (Fig. 7E) [53]. Notably, no significant changes in PCs, PEs, lysophosphatidylcholines, or lysophosphatidylethanolamines were detected (Fig. S25), as well as no upregulation in oxidized lipid species in PDT-treated tumors. To summarize, in vivo lipidomic analysis of PDT-treated OVCAR-3 tumors corroborates the role of Cer signaling in PDT-induced cell killing. Further studies are warranted to comprehensively investigate the ability of PDT to induce lipid peroxidation in vivo.

## Discussion

**Fig. 8:**
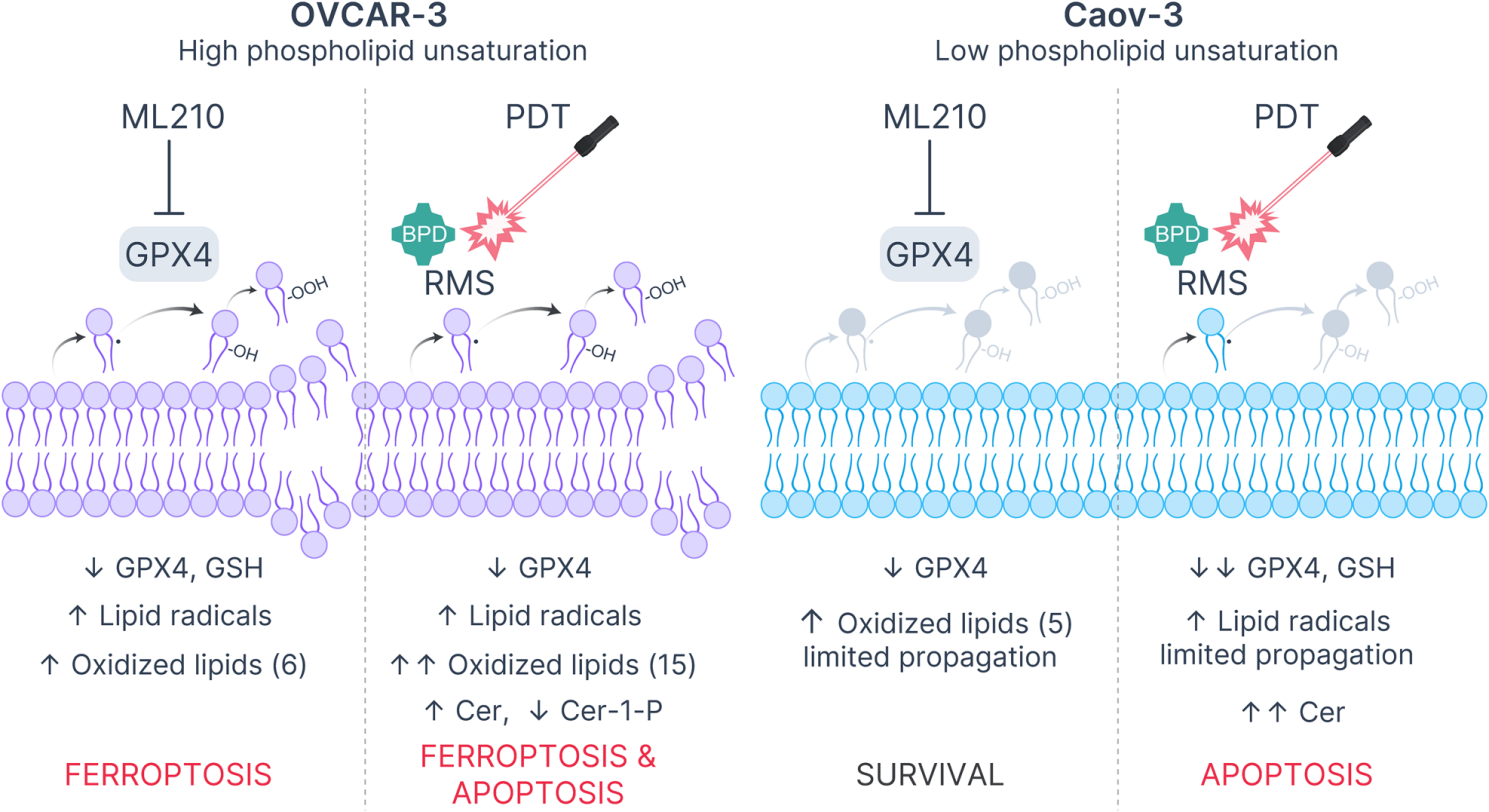
Mechanistic comparison of ML210-induced ferroptosis and photodynamic cell killing in OVCAR-3 and Caov-3 cells based on the underlying lipidomic changes. In cells with relatively high phospholipid unsaturation (left panel), GPX4 inhibition with ML210 unlocks intrinsic enzymatic and non-enzymatic lipid peroxidation, leading to a decrease in the intracellular GSH content, accumulation of lipid radicals and oxidized lipid species, and, eventually, ferroptosis. PDT using BPD results in the formation of lipid radicals, which propagate oxidative damage and lead to the accumulation of oxidized lipids. In cells with relatively low phospholipid unsaturation (right panel), GPX4 inhibition fails to induce critical accumulation of oxidized lipids (depicted in gray). Lipid radicals produced by PDT likely fail to propagate lipid peroxidation due to the lack of unsaturated targets. PDT, however, robustly induces Cer upregulation in both ferroptosis-sensitive and -resistant cell lines, likely contributing to mitochondrial apoptosis.

While mechanisms of photodynamic lipid peroxidation were described decades ago [67, 68], the utility of these effects has only recently come into focus with the discovery of ferroptosis as an effective approach to targeting chemoresistant cells. The localized nature of PDT, along with the availability of clinically viable strategies to deliver light to solid tumors, opened a highly attractive means of inducing lipid peroxidation that does not rely on systemic inactivation of redox defenses (e.g., GPX4 inhibition). The growing interest in this area is reflected in the exponential increase in the number of publications indexed in Scopus that include the terms “photodynamic”, “ferroptosis”, and “cancer” rising from just 1 publication in 2017 to 129 in 2024. Most of these studies focus on the co-delivery of photosensitizers with established small-molecule ferroptosis inducers, often demonstrating impressive synergy [69]. Despite increasing interest in PDT and ferroptosis, the inherent capacity of PDT to induce lipid peroxidation has not been explored with nearly as much enthusiasm. In fact, the specific lipid targets of PDT, the kinetics of photodynamic lipid peroxidation, and the influence of diverse lipidomic landscapes on PDT-lipid interactions largely remain *terra incognita*. This study begins to address this gap by comprehensively interrogating the effects of PDT using a clinical photosensitizer, BPD, on lipid composition and redox states in two ovarian cancer cell lines with distinct lipidomes and ferroptosis sensitivity profiles (Fig. 8).

OVCAR-3 and Caov-3 cells were deliberately chosen as they not only represent primary and metastatic HGSOC, but also have well-documented differences in chemosensitivity, bioenergetic, and antioxidant profiles [59, 70, 71]. Because Caov-3 cells rely heavily on oxidative phosphorylation for ATP production, they also have a relatively high baseline level of RMS. OVCAR-3 cells, on the other hand, use both oxidative phosphorylation and glycolysis, and therefore have a lower baseline level of RMS compared to Caov-3 cells [71, 72]. Relatively high GSH content in Caov-3 cells described in this study may indicate that this cell line relies on GSH to scavenge excess radicals generated due to active oxidative phosphorylation (Fig. S11). Such heightened antioxidant content, along with relatively low phospholipid unsaturation, likely contributes to the observed resistance to both Cum OOH and ML210-induced ferroptosis in Caov-3 cells. Additionally, the significant increase in ubiquinone (coenzyme Q10) content in ML210-treated Caov-3 cells indicates active involvement of ferroptosis suppressor protein 1 in mediating ferroptosis resistance in this cell line (Fig. S22) [13]. Another factor that drives differences in ferroptosis sensitivity is the availability of ferrous iron [73]. While the levels of iron, transferrin receptor 1, and ferroportin were not measured in this study, heightened sensitivity to an iron chelator (DFO) in OVCAR-3 cells (Fig. S19) may indicate their dependence on a relatively high intracellular labile iron pool, which likely facilitates ML210-induced lipid peroxidation via Fenton reaction. Interestingly, while iron chelation with DFO was protective against ML210-induced cell death, it failed to protect cells from PDT-induced cytotoxicity and lipid radical generation. These results indicate that, unlike canonical ferroptosis, PDT-induced lipid peroxidation is less dependent on the intracellular labile iron pool [47].

Despite striking differences in underlying biology and ferroptosis sensitivity, PDT efficacy remained comparable between OVCAR-3 and Caov-3 cells. However, the mechanisms driving this efficacy appear to differ substantially. In ferroptosis-sensitive OVCAR-3 cells, PDT effectively induced lipid radical generation, as evidenced by shifts in the oxidized/unoxidized C11 BODIPY fluorescence ratio and the emergence of a diverse array of oxidized lipid species identified by redox lipidomics. Notably, 4 out of 6 oxidized lipids upregulated by ML210 were also found in PDT-treated cells, indicating substantial mechanistic overlap between PDT and canonical ferroptosis (Fig. 5A, B). In contrast, although PDT initiated lipid radical formation in Caov-3 cells, it failed to yield comparable accumulation of oxidized species. This discrepancy may stem from lower phospholipid unsaturation, slowing lipid radical propagation, enhanced antioxidant defenses (e.g., higher GSH levels), or differences in cell death kinetics. Consistent with these observations, Lip-1 provided partial protection from PDT-induced cell death in both cell lines, though the rescue was more pronounced in OVCAR-3. This suggests that lipid radical formation contributes to PDT cytotoxicity in both cell lines but plays a more dominant role in ferroptosis-prone OVCAR-3 cells due to their higher susceptibility to lipid peroxidation and potentially weaker redox buffering capacity.

The differences in OVCAR-3 and Caov-3 response to PDT are also reflected in their respective GPX4-GSH antioxidant responses. Treatment with ML210 decreased GPX4 protein concentration in both cell lines compared to the control. This likely indicates that the formation of ML210-GPX4 adducts results in conformational changes that prevent its recognition by antibodies. While ML210 decreased GPX4 concentration to a similar extent in both cell lines, it did not induce significant cell killing in Caov-3 cells. Similarly, PDT decreased GPX4 protein concentration, albeit not significantly in OVCAR-3 cells, indicating that the RMS produced during PDT may induce GPX4 degradation. Although changes in GPX4 concentrations in response to PDT and ML210 were quite consistent between these cell lines, OVCAR-3 and Caov-3 cells showed opposite responses with respect to GSH concentration. While ML210 induced a significant decrease in GSH content in OVCAR-3 cells, PDT induced little to no change in this cell line. Together with their lower baseline GSH levels compared to Caov-3 cells, such a lack of GSH decrease may suggest the use of alternative antioxidant mechanisms in these relatively chemoresistant cells [74]. Caov-3 cells, on the other hand, showed little change in GSH concentration in response to ML210, but PDT induced a significant and dose-dependent decrease in GSH concentration.

In both cell lines, however, PDT induced significant and dose-dependent upregulation of Cer. Cer play a key role in mitochondrial membrane permeabilization leading to cytochrome c leakage [75], and de novo Cer synthesis in the ER is a critical prerequisite for PDT-induced mitochondrial apoptosis [52, 53]. Importantly, the involvement of Cer signaling in PDT response was strongly supported by our in vivo data. Tumor tissues exhibited a robust increase in Cer levels accompanied by a marked decrease in Cer-1-P, a bioactive sphingolipid known to promote cell survival and inflammation [54]. This shift suggests a substantial reprogramming of the “ceramide shunt” from a pro-survival toward a pro-apoptotic phenotype following PDT [54]. While PDT-induced Cer accumulation has been documented previously [52, 53], to our knowledge, this is the first report of a concomitant reduction in Cer-1-P after PDT, in vitro or in vivo. Additionally, we observed increased levels of Hex2Cer (both in vitro and in vivo), a glycosylated Cer derivative with reduced pro-apoptotic potency [76]. This may represent a compensatory response aimed at buffering Cer accumulation and limiting apoptosis. Notably, overexpression of UDP-glucose ceramide glucosyltransferase (UGCG), which converts Cer into glucosylceramide and downstream glycosphingolipids including Hex2Cer, has been associated with enhanced proliferation and chemoresistance in multiple cancers [77, 78]. Moreover, previous studies have shown that pharmacologic inhibition of acid ceramidase with LCL521 augments PDT efficacy by increasing dihydroceramide accumulation [79]. Together, these findings highlight the therapeutic relevance of the Cer–glycosphingolipid axis in modulating PDT response. Targeting Cer clearance pathways, such as UGCG or ceramidase, may therefore offer a rational strategy to potentiate pro-apoptotic signaling and improve PDT outcomes.

As lipid and metabolism-targeting interventions gain recognition for overcoming chemoresistance in HGSOC, PDT is emerging as a versatile tool. It can directly induce lipid peroxidation and indirectly alter lipid composition, for example, by enhancing Cer synthesis. Furthermore, given that Cer have emerged as damage-associated molecular patterns (DAMPs) [80], the ability of PDT to upregulate these lipids in vitro and in vivo warrants further investigation as a potential strategy to modulate the tumor immune microenvironment and improve outcomes of complementary immunotherapies. In this study, we (1) demonstrated the robust efficacy of PDT across cell lines with distinct chemotherapy and ferroptosis sensitivity profiles, (2) identified lipid oxidation targets shared between PDT and canonical ferroptosis, and (3) mapped PDT-induced lipidomic rearrangements, most notably the dose-dependent upregulation of apoptosis-inducing ceramides. In the context of HGSOC, further studies are needed to explore the potential of PDT-induced lipid oxidation and compositional changes to enhance the efficacy of standard-of-care chemotherapy. Beyond HGSOC, structure-activity relationship studies and formulation development efforts are warranted to design photodynamic agents specifically tailored to promote lipid peroxidation and ferroptosis, targeting a range of chemoresistant cancers where ferroptosis has proven effective.

## Supplementary information

Supplementary information

## Data Availability

All supporting data and relevant analyses are available at UNC Dataverse, a public data repository service for the University of North Carolina at Chapel Hill (UNC) research community, and can be accessed using the following link: https://dataverse.unc.edu/dataverse/Overchuketal_CDDis_2025.
