# Supplementary material for: Photodynamic therapy simultaneously induces ferroptosis- and apoptosis-like lipid signatures in ovarian cancer cells
Source: Cell Death Dis. 2025 Dec 10;16(1):899. doi: 10.1038/s41419-025-08189-5 (PMC12722221; doi:10.1038/s41419-025-08189-5)
Supplement: Supplementary file 1 — Supplementary information [file 41419_2025_8189_MOESM1_ESM.docx]

**Supplementary Materials**


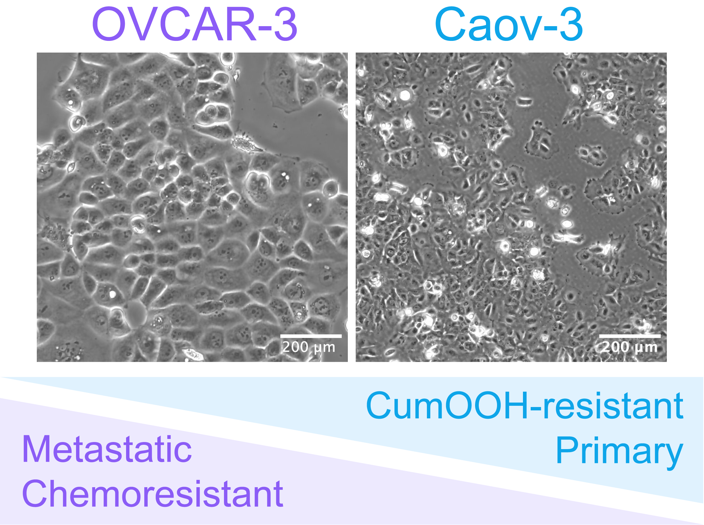


**Figure S1.** Representative phase contrast microscopy images of relatively chemoresistant OVCAR-3 cells and primary and relatively chemosensitive Caov-3 cells. Scale bar = 200 μm.

**
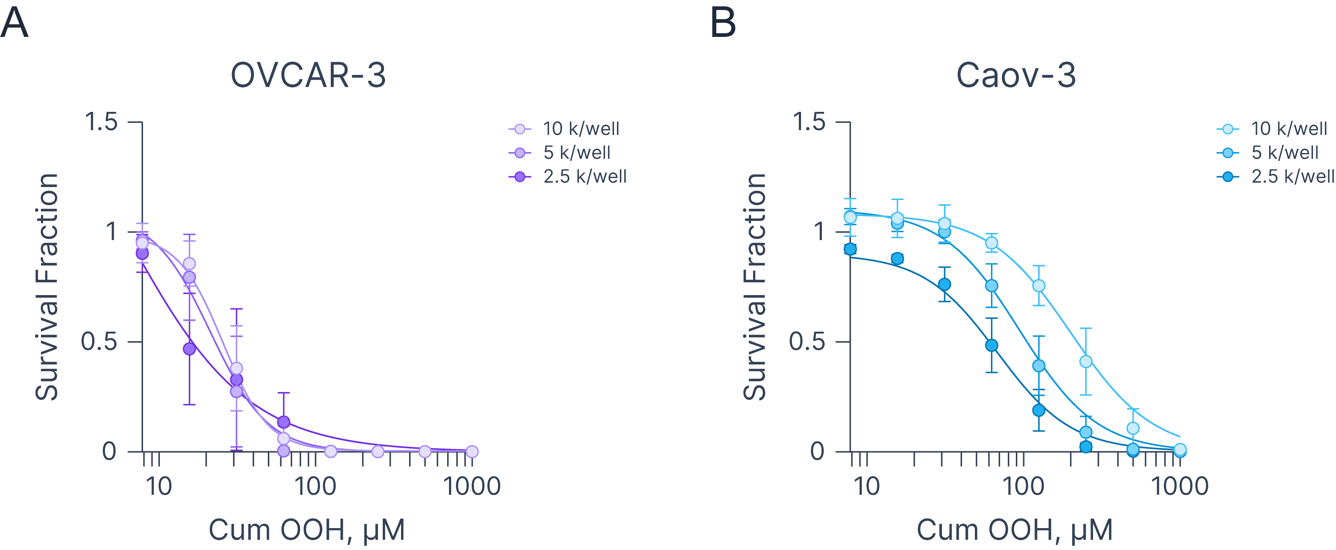
**

**Figure S2.** Cumene hydroperoxide (Cum OOH) dose–response curves in **(A)** OVCAR-3 and **(B)** Caov-3 cells seeded at densities of 0.25 × 10^4^, 0.5 × 10^4^, and 1 × 10^4^ cells/well (2.5, 5, and 10 k/well). Each data point represents the mean of three independent biological replicates, each performed in duplicate. Error bars indicate the standard error of the mean. Curve fitting was performed using four-parameter non-linear regression in Graphmatik (v. 0.3.2).

**
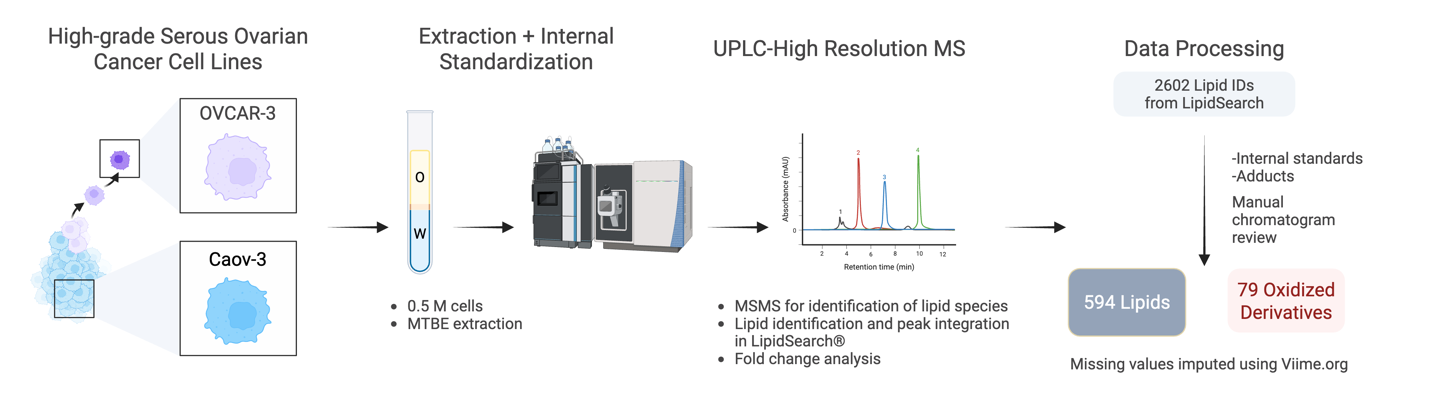
**

**Figure S3.** Experimental workflow and data processing of lipidomic experiments.

**
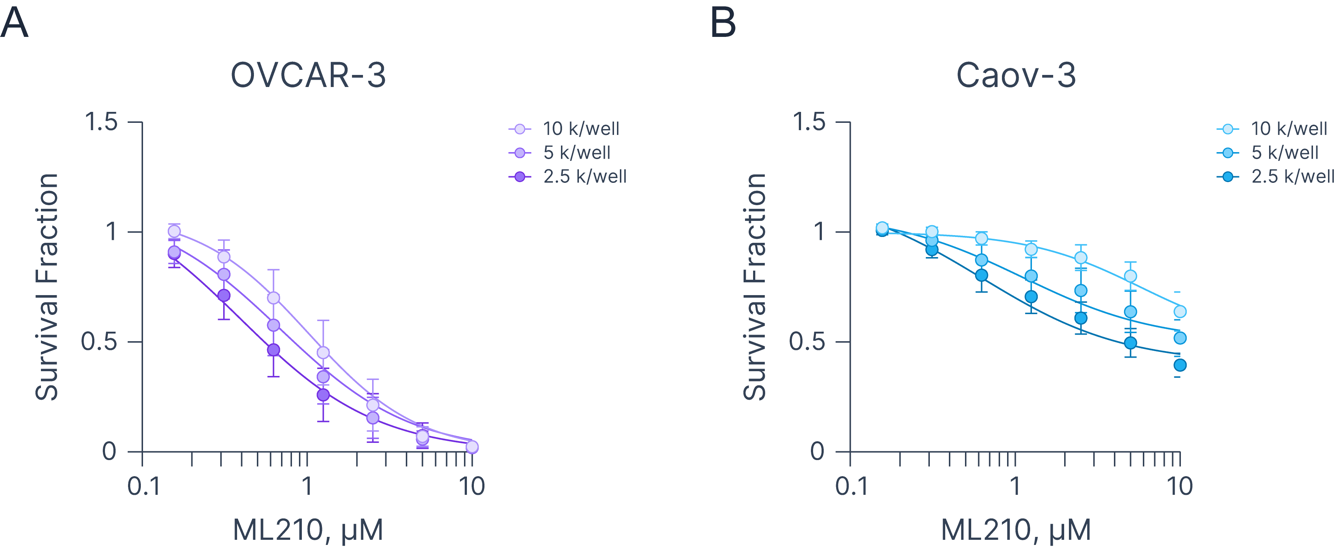
**

**Figure S4.** ML210 dose–response curves in OVCAR-3 **(A)** and Caov-3 **(B)** cells seeded at densities of 0.25 × 10^4^, 0.5 × 10^4^, and 1 × 10^4^ cells/well. Each data point represents the mean of seven independent biological replicates, each performed in duplicate. Error bars indicate the standard error of the mean. Curve fitting was conducted using four-parameter non-linear regression in Graphmatik (v. 0.3.2).


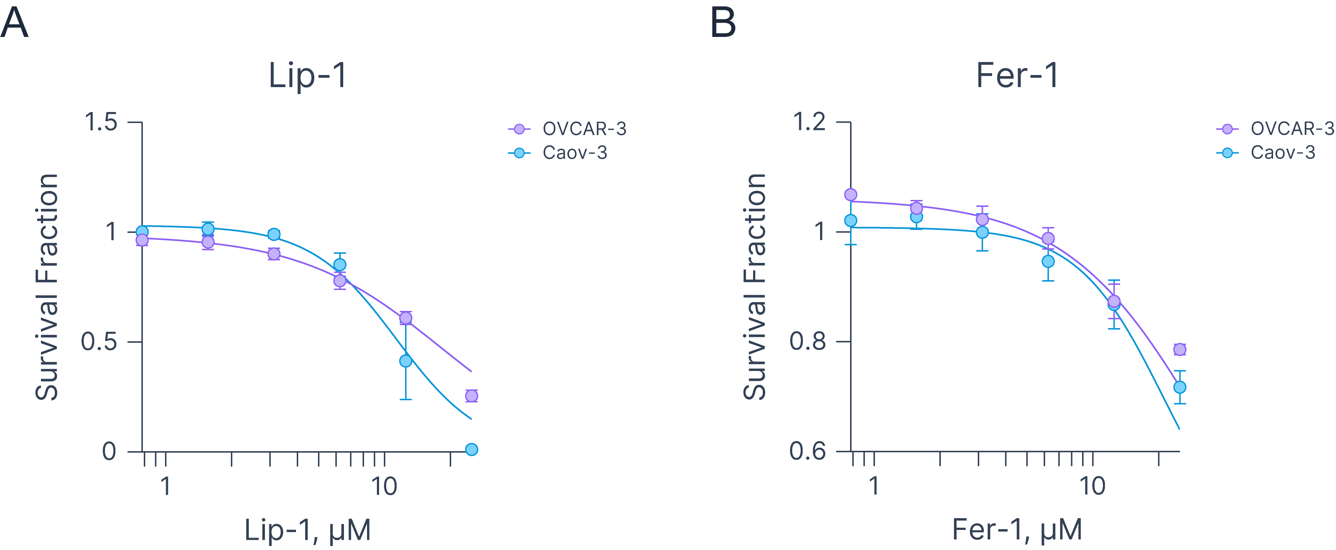


**Figure S5.** Survival fractions in OVCAR-3 and Caov-3 cells treated with escalating concentrations of **(A)** liproxstatin-1 (Lip-1) and **(B)** ferrostatin-1 (Fer-1) for 72 h. Statistics: Each point represents a mean of four independent biological replicate each containing at least two replicates. Error bars represent the standard error of the mean. Curve fitting was conducted using four-parameter non-linear regression in Graphmatik (v. 0.3.2).

**Figure S6.** Pharmacological inhibition of ML210-induced ferroptosis in OVCAR-3 and Caov-3 cells at 2.5k cells/well **(A, B),** 5k cells/well **(C, D)** and 10k cells/well **(E, F).** Each point represents a mean of an independent biological replicate each containing at least two technical replicates. Error bars represent the standard error of the mean. Two-way ANOVA with Šidák correction for multiple comparisons, *p ≤ 0.05, **p ≤ 0.01, ***p ≤ 0.001, ****p ≤ 0.0001, performed in GraphPad Prism v. 10.4.0.

**Figure S7.** Survival fractions in OVCAR-3 and Caov-3 cells treated with buthionine sulphoximine (BSO) at 0-500 μM for 6 h. Each point represents a mean of three independent biological replicates each containing three technical replicates. Error bars represent the standard error of the mean.

**Figure S8.** Survival fractions in OVCAR-3 and Caov-3 cells treated with BSO (500 μM) and ML210 (2.5 μM) for 6 h. Each point represents a mean of an independent biological replicate each containing two replicates. Error bars represent the standard error of the mean. One-way ANOVA with Dunnett’s correction for multiple comparisons, **p<0.01, performed in GraphPad Prism v. 10.4.0.

**Figure S9.** Glutathione (GSH) content in untreated OVCAR-3 and Caov-3 cells normalized by ATP-based CellTiter-Glo assay. Each point represents a mean of an independent biological replicate each containing two technical replicates. Error bars represent the standard error of the mean. Unpaired t-test, **p<0.01, performed in GraphPad Prism v. 10.4.0.


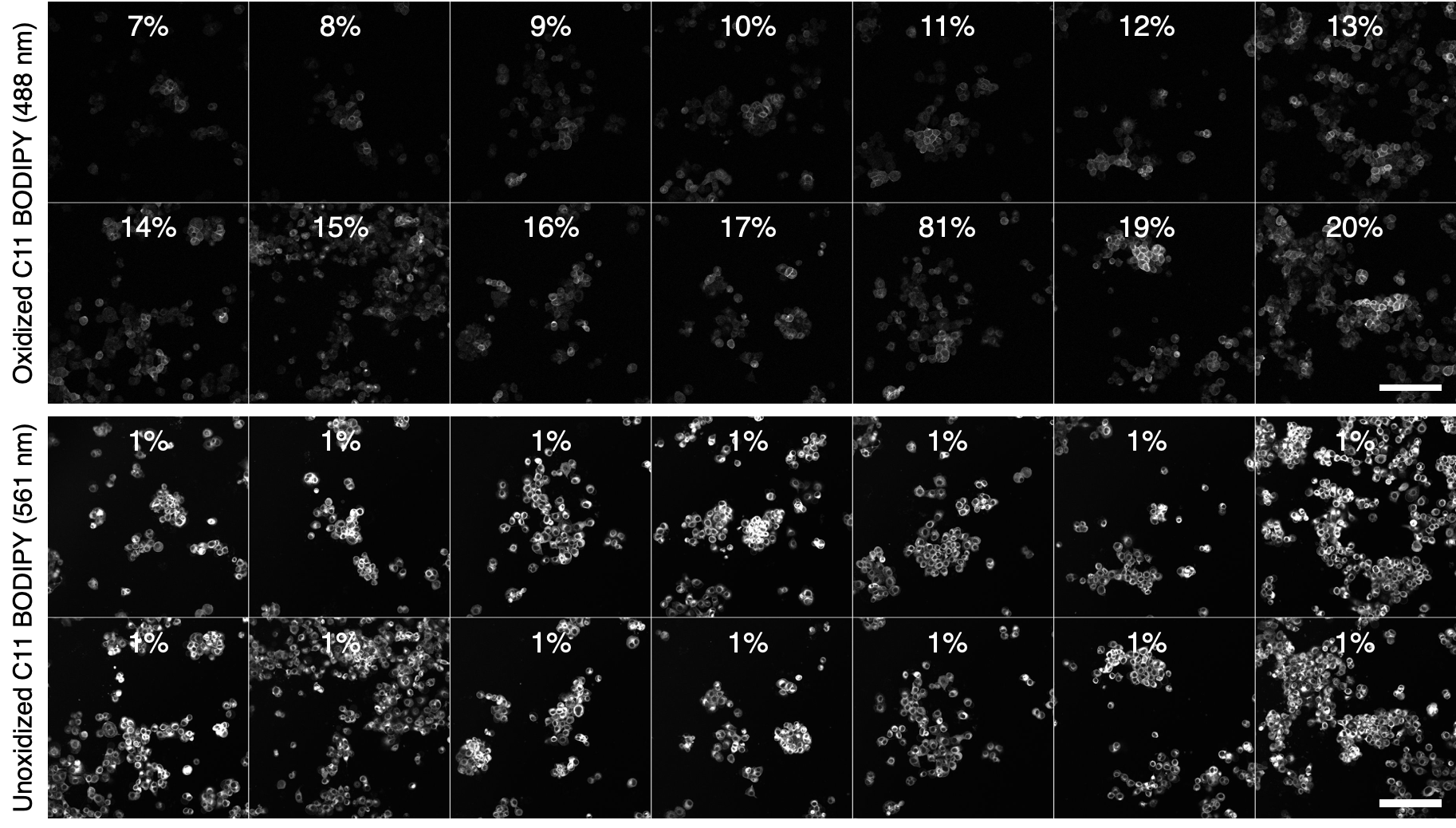


**Figure S10.** Increasing fluorescence of oxidized C11 BODIPY at escalating 488 nm laser power. OVCAR-3 cells treated with 2.5 μM ML210 for 6 h were stained with 10 μM C11 BODIPY for 30 min. Oxidized C11 BODIPY fluorescence signal increased as the 488 nm laser power was escalated from 7% to 20%. Unoxidized C11 BODIPY signal was captured at 1% 561 nm laser power.


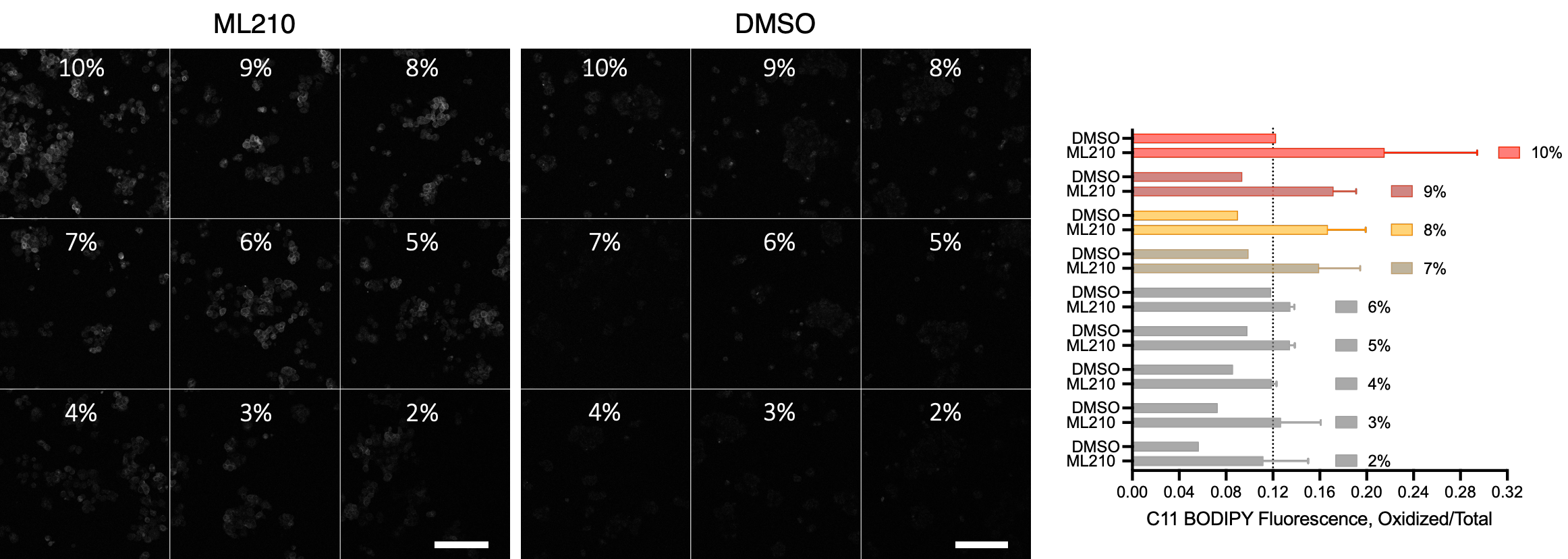


**Figure S11.** To determine the lowest 488 nm laser power that maintains an acceptable signal-to-noise ratio, OVCAR-3 cells treated with 2.5 μM ML210 for 6 hours were stained with 10 μM C11 BODIPY for 30 minutes. Oxidized C11 BODIPY was imaged using 488 nm laser at 2–10% power, while unoxidized C11 BODIPY was imaged at 1% laser power (images not shown). The ratio of oxidized to total C11 BODIPY fluorescence was consistently higher in ML210-treated samples compared to DMSO-treated samples at 7–10% laser power (signal-to-noise >1.5). Consequently, 7% 488 nm laser power was selected for use in subsequent experiments as it would result in the least amount of photobleaching, while maintaining an acceptable signal-to-noise ratio.

**Figure S12.** Survival fractions in OVCAR-3 (**A, C**) and Caov-3 (**B, D**) cells 72 h treated with escalating concentrations of Lip-1 and Fer-1 for 1.5 h. This time interval was selected to mimic inhibitor co-incubation with BPD during PDT +/- inhibition experiments. Incubation with 10 μM of Lip-1 or Fer-1 did not induce cytotoxicity in either cell line (B, D). A, C: Each point represents a mean of at least three biological replicates, each performed in duplicate. Error bars indicate the standard error of the mean. Curves represent non-linear "[Inhibitor] vs. response -- Variable slope (four parameters)" fit of inhibitor dose-responses in OVCAR-3 and Caov-3 cells performed in GraphPad Prism v. 10.4.0. B, D: Each point represents a mean of an independent biological replicate each containing at least two replicates. Error bars indicate the standard error of the mean. One-way ANOVA with Dunnett’s correction for multiple comparisons performed in GraphPad Prism v. 10.4.0.


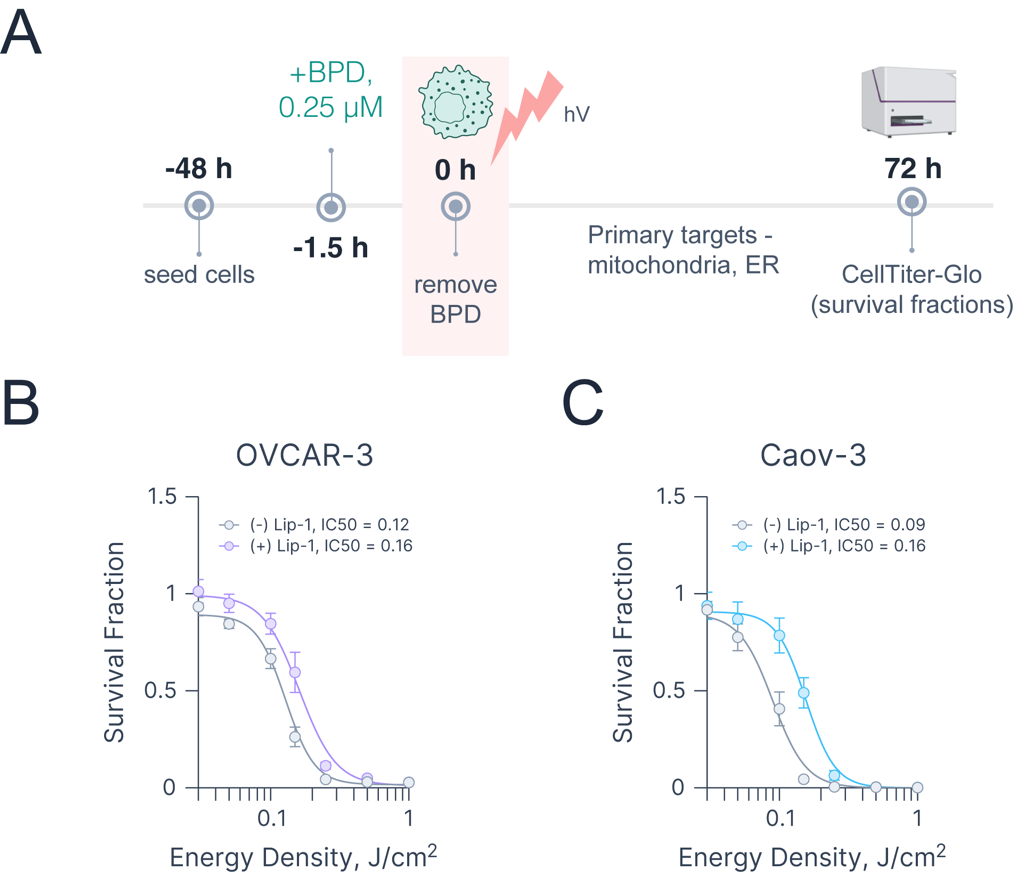


**Figure S13.** Workflow schematic illustrating the conventional PDT protocol in OVCAR-3 and Caov-3 cells, in which 0.25 μM BPD is administered 90 min prior to light irradiation, allowing internalization and redistribution to mitochondria and endoplasmic reticulum (ER) **(A).** PDT dose–response curves in the presence or absence of 10 μM Lip-1 in OVCAR-3 **(B)** and Caov-3 **(C)** cells. Statistics: Each point represents the mean of an independent biological replicate, with each replicate containing two technical replicates. Error bars indicate the standard error of the mean. Lines represent a four-parameter nonlinear regression fit, R squared > 0.99 for both. OVCAR-3: IC50 = 0.12 (–Lip-1), IC50 = 0.16 (+Lip-1), R squared > 0.99 for both. Caov-3: IC50 = 0.09 (–Lip-1), IC50 = 0.16 (+Lip-1). All graphs were generated using Graphmatik (ver. 0.3.2, 2025).


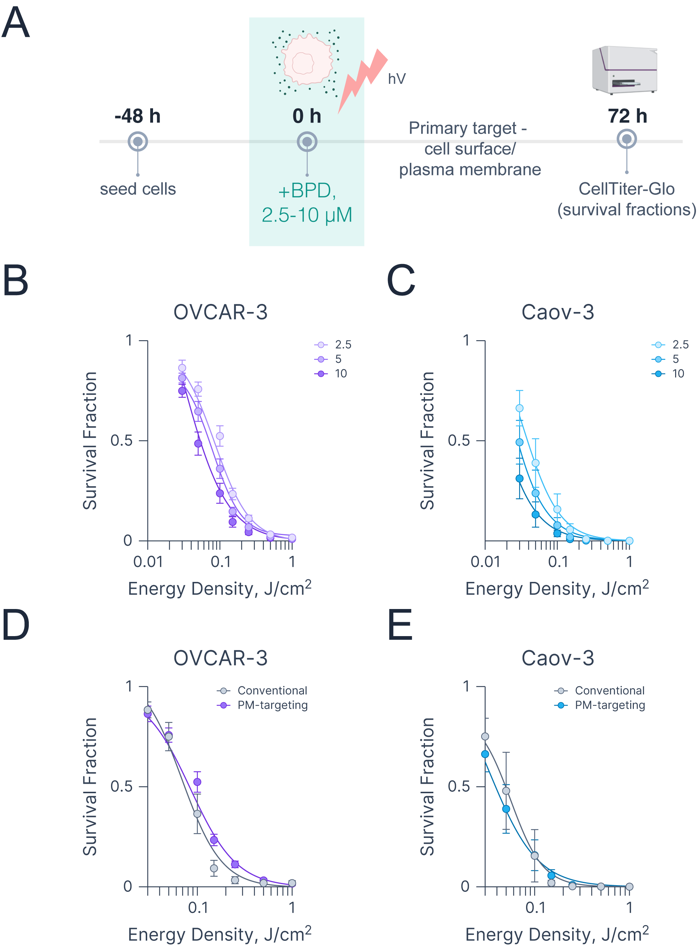


**Figure S14.** Workflow schematic illustrating short drug-light interval PDT (SDLI-PDT) in OVCAR-3 and Caov-3 cells **(A).** BPD at 2.5, 5, or 10 μM was added immediately prior to light irradiation. Dose–response curves for SDLI-PDT in OVCAR-3 **(B)** and Caov-3 **(C)** cells exposed to 2.5, 5, or 10 μM BPD during light exposure. Comparison of SDLI-PDT (2.5 μM BPD) with conventional PDT targeting mitochondria and endoplasmic reticulum (0.25 μM BPD, 90 min incubation time) in OVCAR-3 **(D)** and Caov-3 **(E)** cells. Statistics: Each dot represents the mean of three biological replicates, each conducted in duplicate. Error bars indicate the standard error of the mean. Lines represent a four-parameter nonlinear regression fit, R squared > 0.99. All graphs were generated using Graphmatik (ver. 0.3.2, 2025).


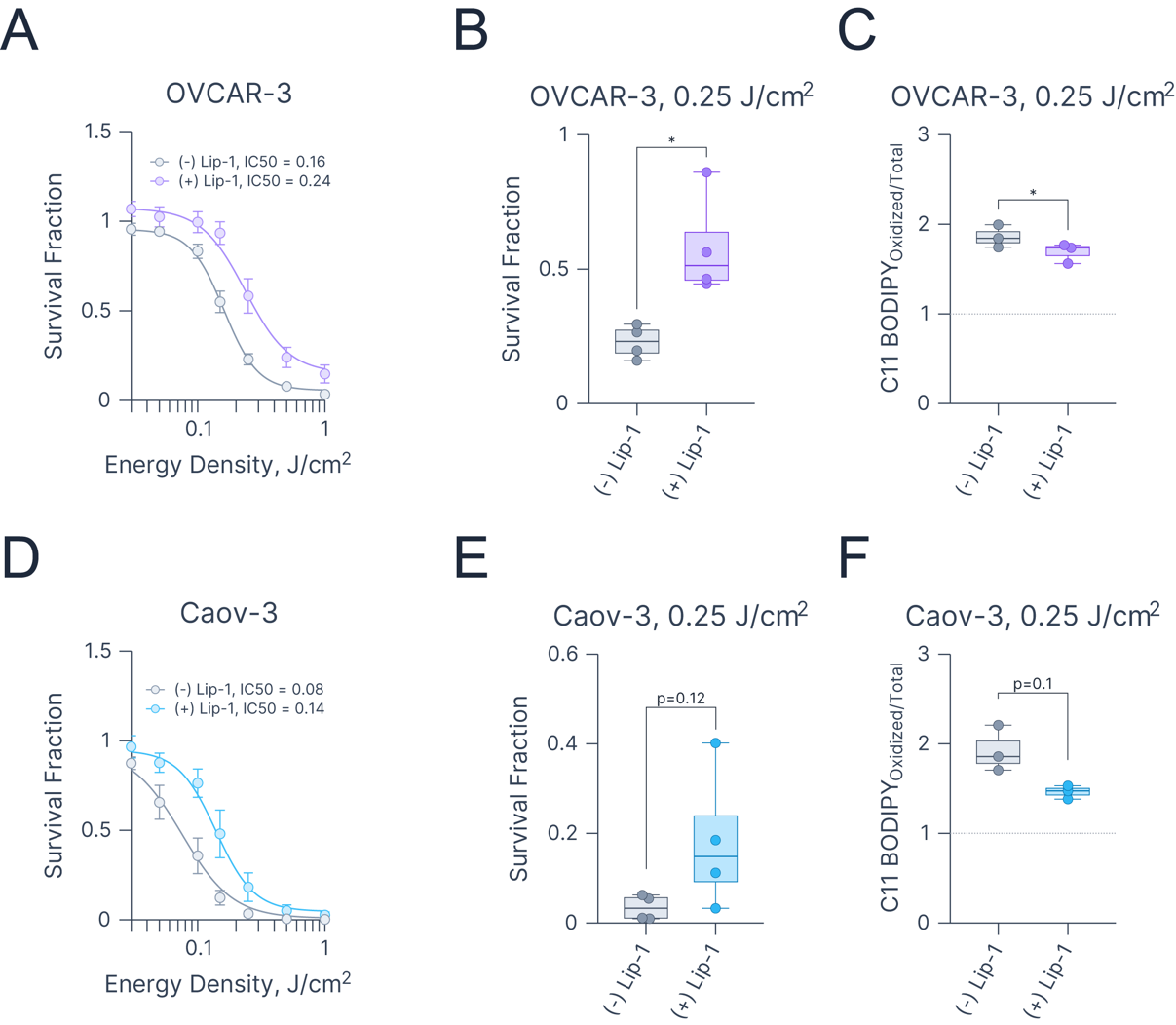


**Figure S15.** Partial rescue from SDLI-PDT-induced cell death by 10 μM Lip-1 in OVCAR-3 cells **(A, B).** Lipid radical levels 24 h post SDLI-PDT (0.25 J/cm^2^) with or without Lip-1 in OVCAR-3 cells **(C).** Partial rescue by 10 μM Lip-1 in Caov-3 cells **(D, E).** Lipid radical levels 24 h post SDLI-PDT with or without Lip-1 in Caov-3 cells **(F).** Statistics: A, D: Each dot represents the mean of four biological replicates, each conducted in duplicate. Error bars indicate the standard error of the mean. Lines represent a four-parameter nonlinear regression fit, R squared > 0.99 for both. OVCAR-3: IC50 = 0.16 (–Lip-1), IC50 = 0.24 (+Lip-1), Caov-3: IC50 = 0.08 (–Lip-1), IC50 = 0.14 (+Lip-1). B, C, E, F: Each dot represents a biological replicate conducted in duplicates, whiskers indicate the range. Statistical significance determined using t-test, *p < 0.05. All graphs were generated in Graphmatik (ver. 0.3.2, 2025).

**Figure S16.** Survival fractions in OVCAR-3 **(A)** and Caov-3 **(B)** cells 6 h after being treated with 0.25 μM BPD in the dark, and PDT at 0.05, 0.25, and 1 J/cm^2^. Statistics: Each point represents a mean of an independent biological replicate each containing two technical replicates. Error bars indicate the standard error of the mean. One-way ANOVA with Dunnett’s correction for multiple comparisons, ***p<0.001, ****p<0.0001, performed in GraphPad Prism v. 10.4.0.


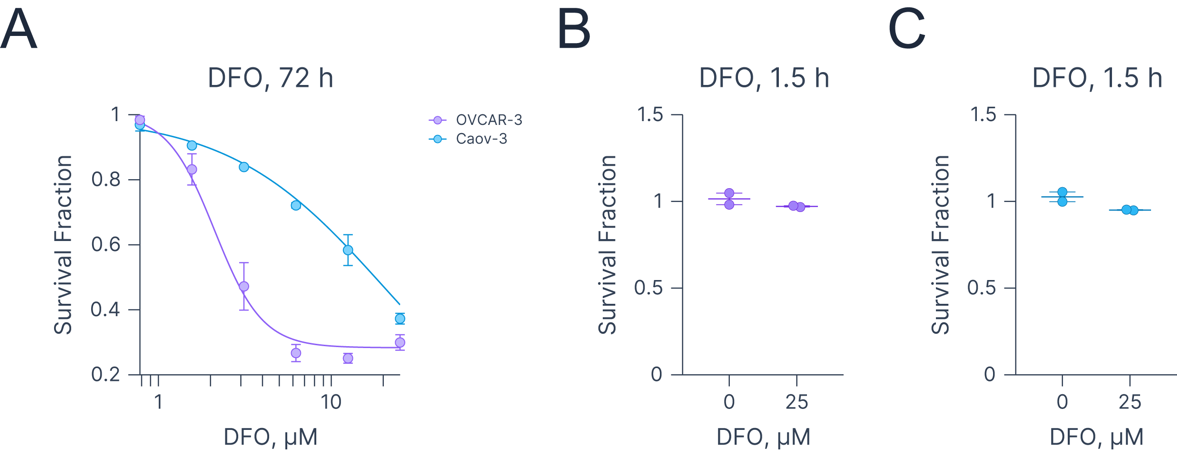


**Figure S17.** Deferoxamine (DFO) dose-response at 72 h incubation **(A)** and 1.5 h incubation mimicking pre-PDT exposure in **(B)** OVCAR-3 and **(C)** Caov-3 cells. Statistics: A: Each point represents the mean of three or more biological replicates each performed in duplicate. Lines represent a four-parameter nonlinear regression fit, R squared > 0.99 for both. Error bars indicate the standard error of the mean. B, C: Each dot represents the mean of a biological replicate each performed in duplicate. Error bars represent standard error of the mean. All graphs were generated in Graphmatik (ver. 0.3.2, 2025).


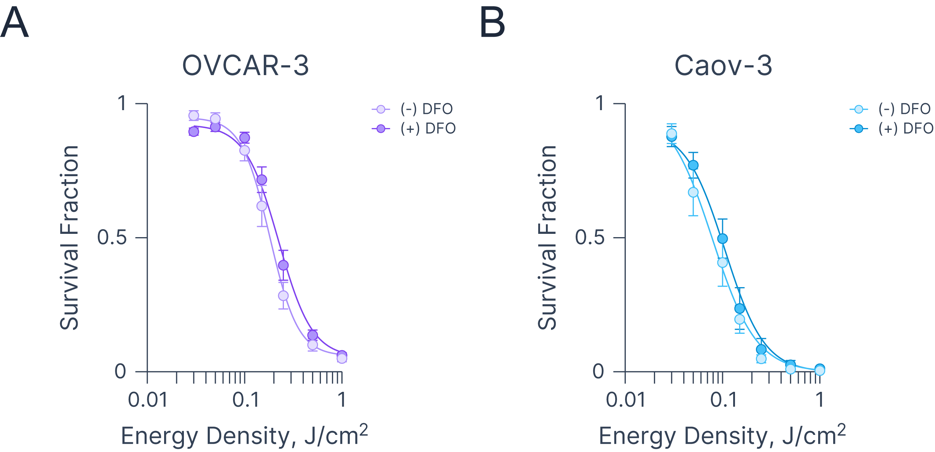


**Figure S18.** SDLI-PDT with or without a 1.5-h pre-incubation with 25 μM DFO in **(A)** OVCAR-3 and **(B)** Caov-3 cells. Statistics: A: Each point represents the mean of three or more biological replicates each performed in duplicate. Error bars indicate the standard error of the mean. Lines represent a four-parameter nonlinear regression fit, R squared > 0.99 for both. All graphs were generated using Graphmatik (ver. 0.3.2, 2025).


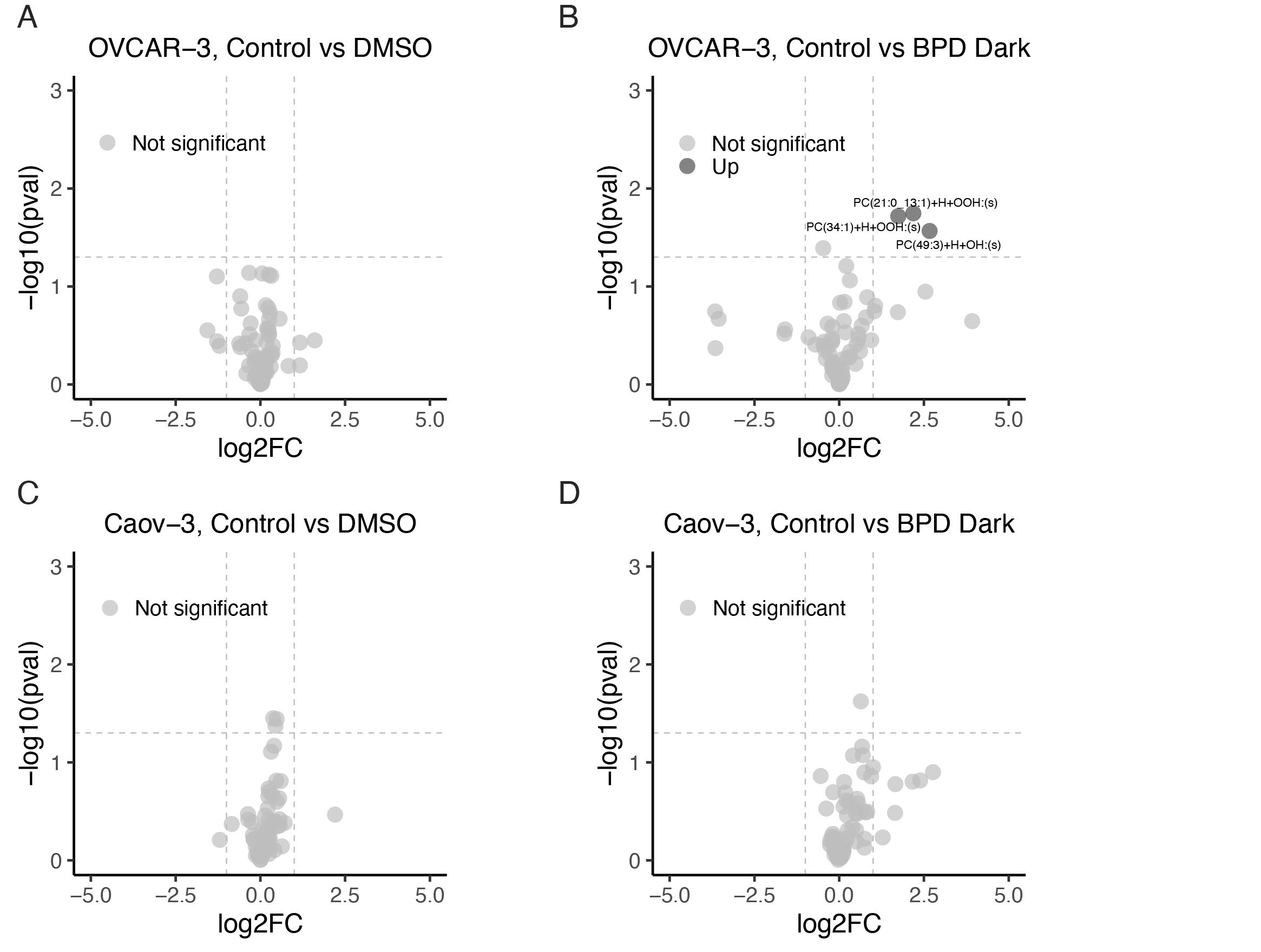


**Figure S19.** Volcano plots of oxidized lipid species. **(A)** OVCAR-3 cells, control versus DMSO, **(B)** OVCAR-3 cells, control versus BPD dark, **(C)** Caov-3 cells, control versus DMSO, **(D)** Caov-3 cells, control versus BPD dark.


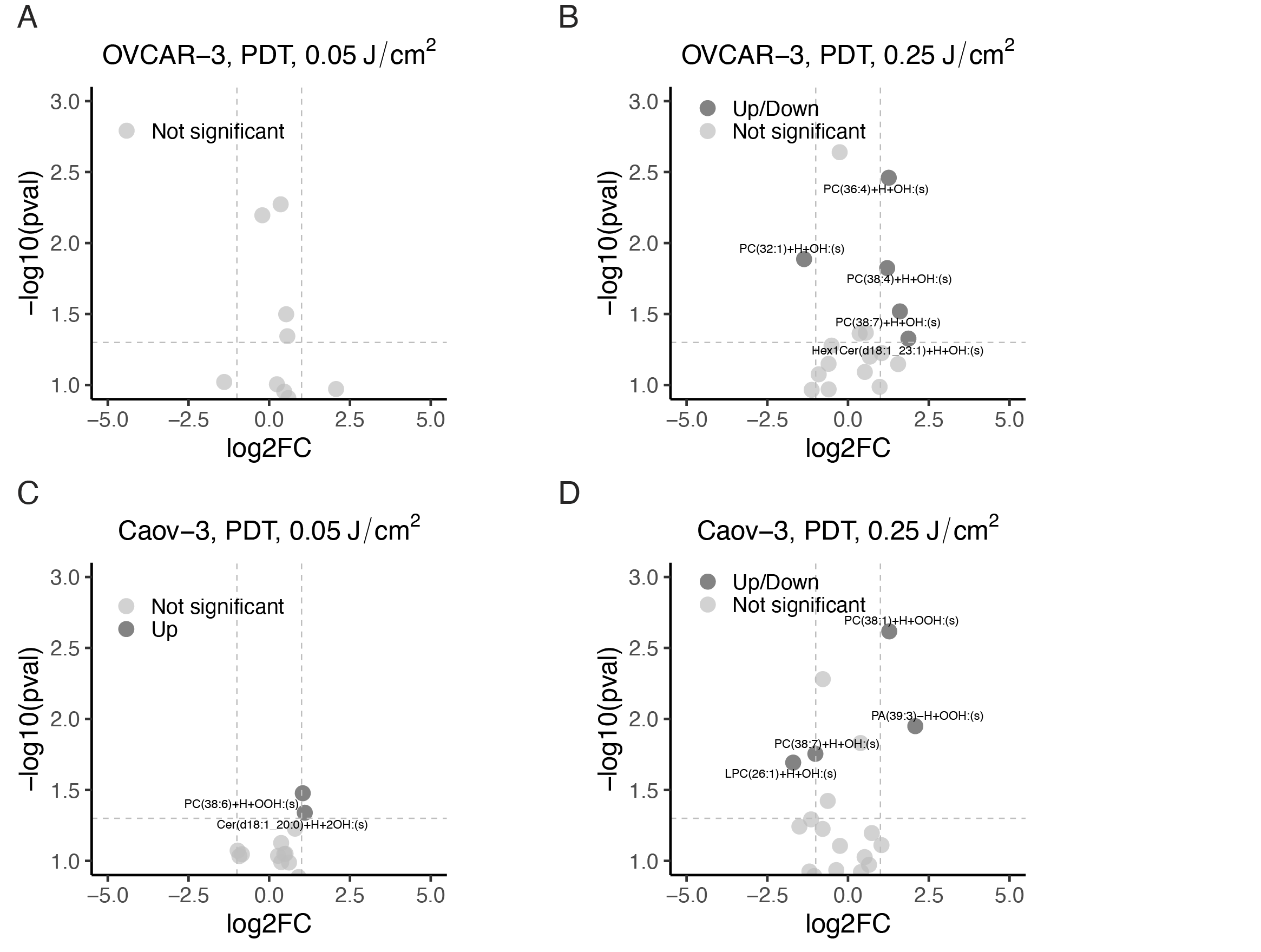


**Figure S20.** Volcano plots of oxidized lipid species in cells treated with PDT at 0.05 and 0.25 J/cm^2^. **(A)** OVCAR-3 cells, BPD dark versus PDT at 0.05 J/cm^2^, **(B)** OVCAR-3 cells, BPD dark versus PDT at 0.25 J/cm^2^, **(C)** Caov-3 cells, BPD dark versus PDT at 0.05 J/cm^2^, **(D)** Caov-3 cells, BPD dark versus PDT at 0.25 J/cm^2^.


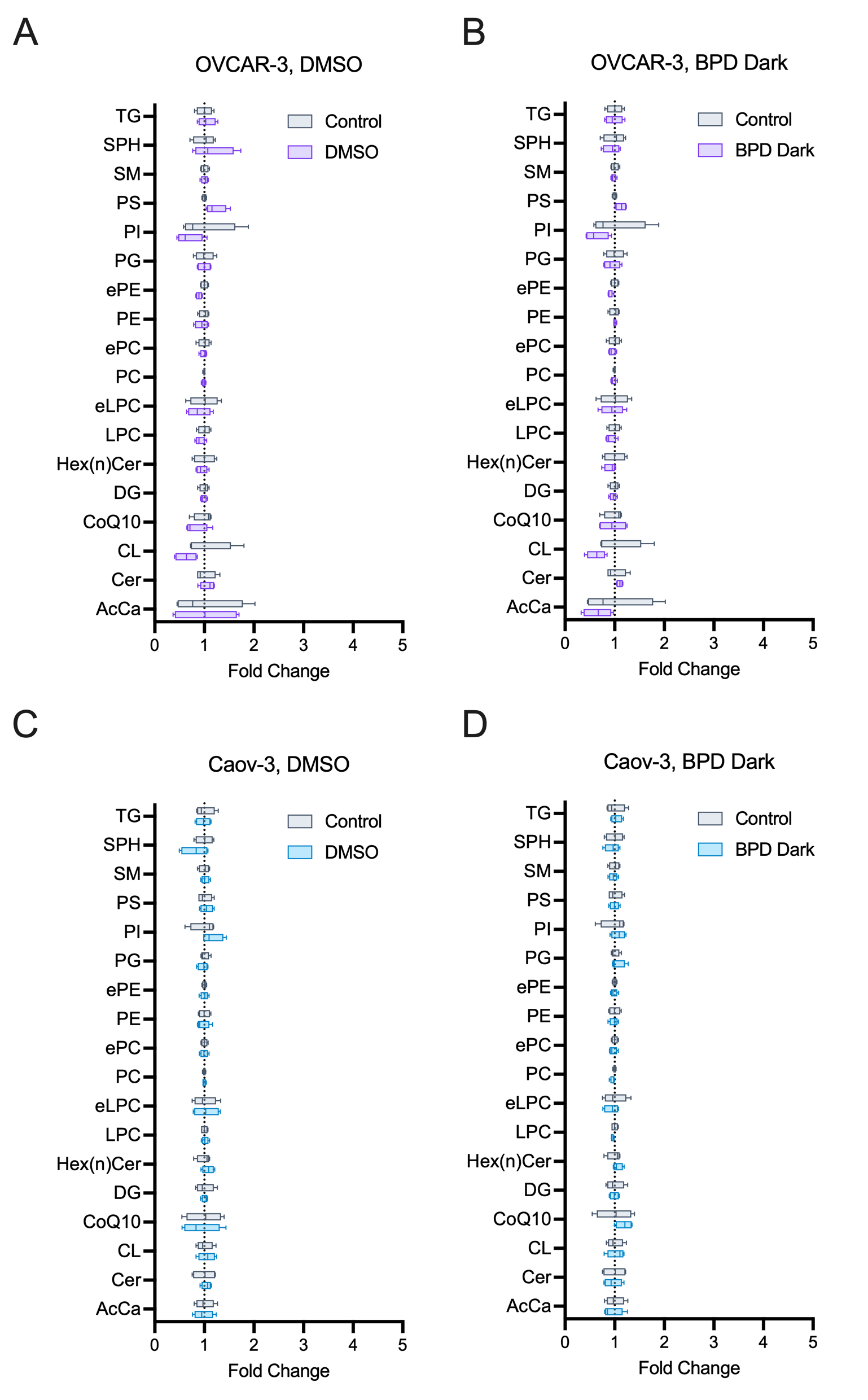


**Figure S21.** Changes in abundances of lipid classes (fold change of total peak areas) upon DMSO and BPD treatment in OVCAR-3 **(A, B)** and Caov-3 cells **(C, D)**. Whiskers represent range.

**
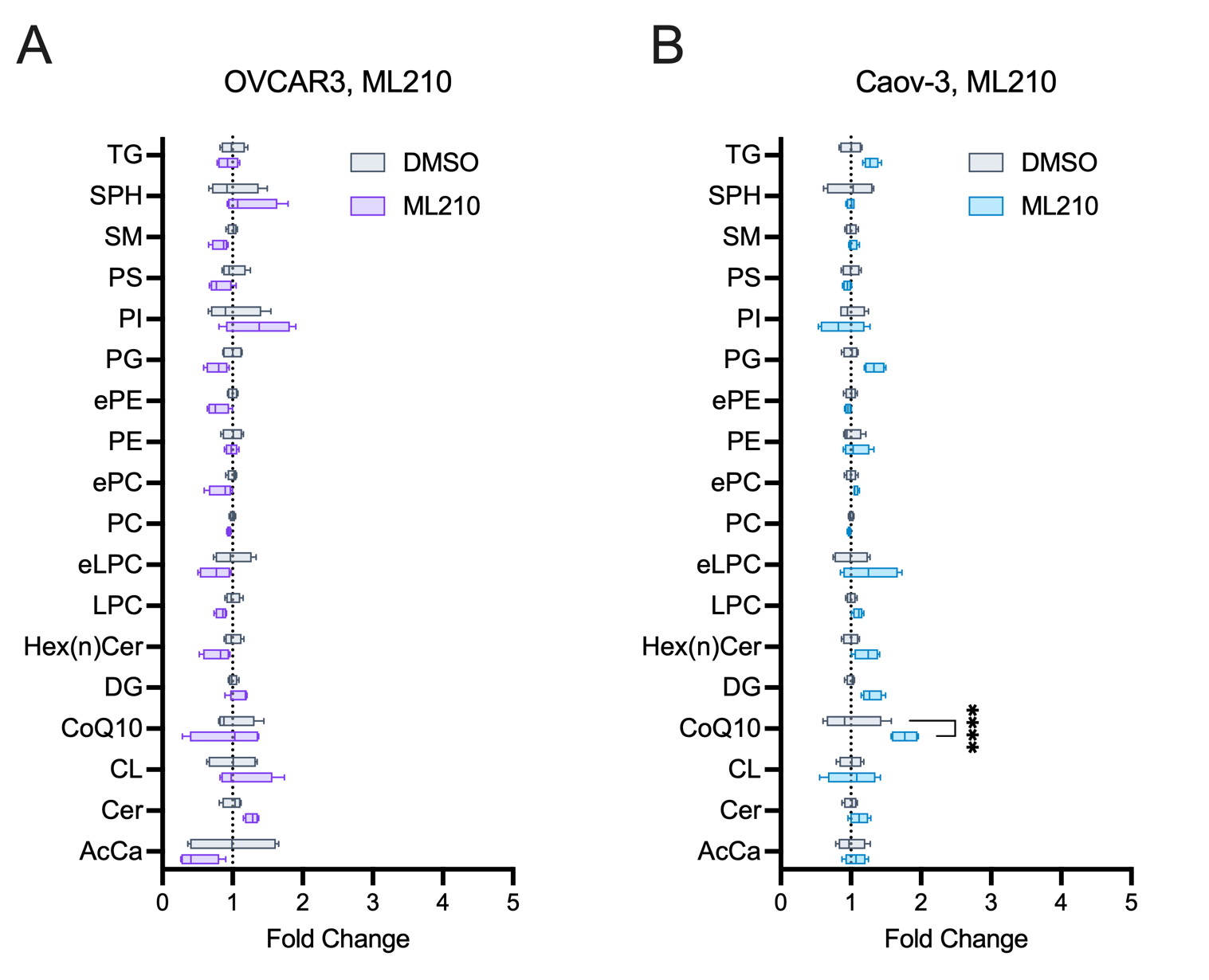
**

**Figure S22.** Changes in abundances of lipid classes (fold change of total peak areas) in ML210-treated OVCAR-3 **(A)** and Caov-3 **(B)** cells. Whiskers represent the range. Two-way ANOVA with Šídák's correction for multiple comparisons, ****p<0.0001. Plots were generated in Graphmatik (Version 0.3.2, 2025).

**
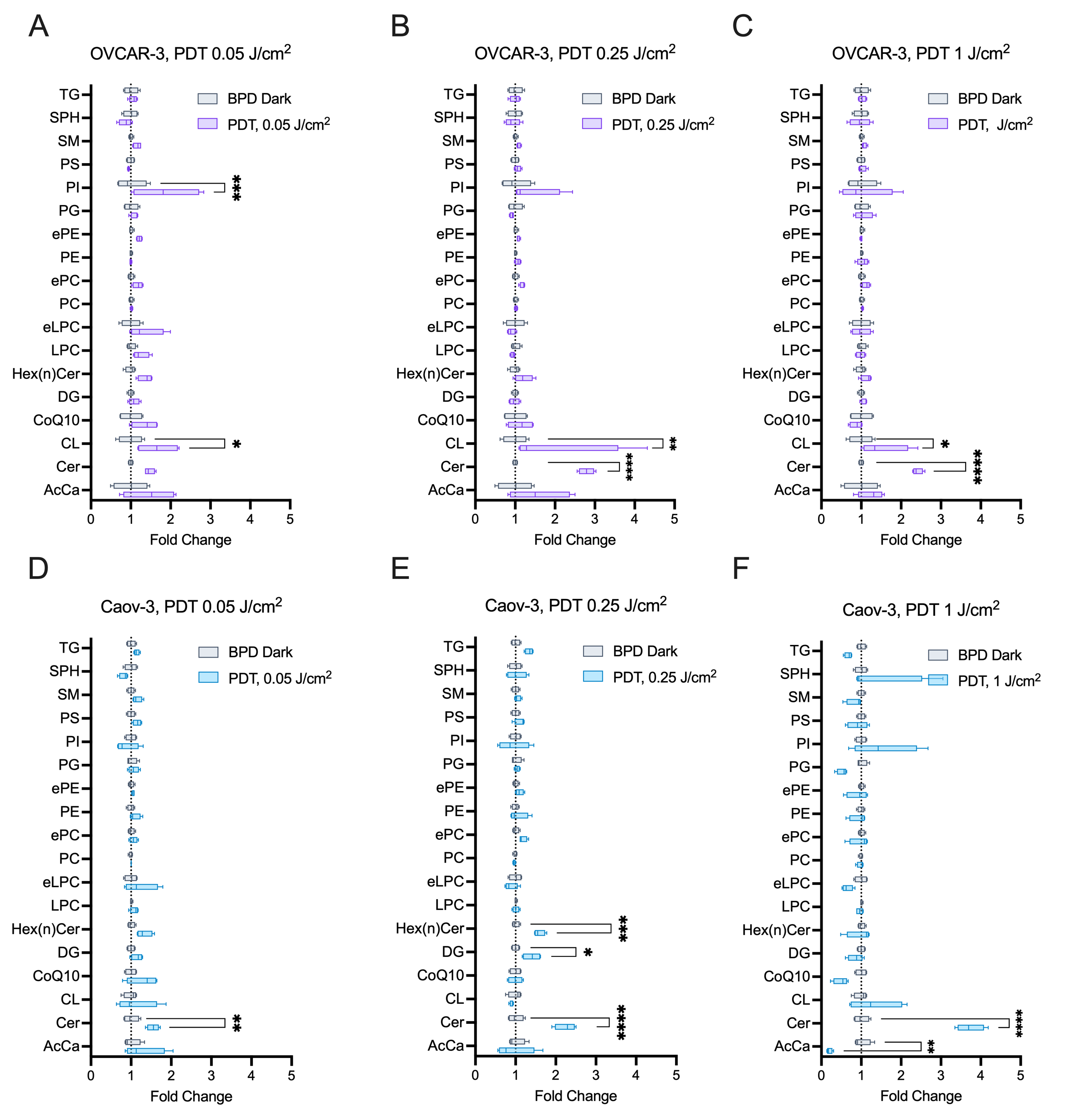
**

**Figure S23.** Changes in abundances of lipid classes (fold change of total peak areas) in OVCAR-3 **(A, B, C**) and Caov-3 **(D, E, F)** cells treated with PDT at 0.05, 0.25, and 1 J/cm^2^. Whiskers represent the range. Two-way ANOVA with Šídák's correction for multiple comparisons, *p<0.05, **p<0.01, ***p<0.001, ****p<0.0001. Plots were generated in Graphmatik (Version 0.3.2, 2025).


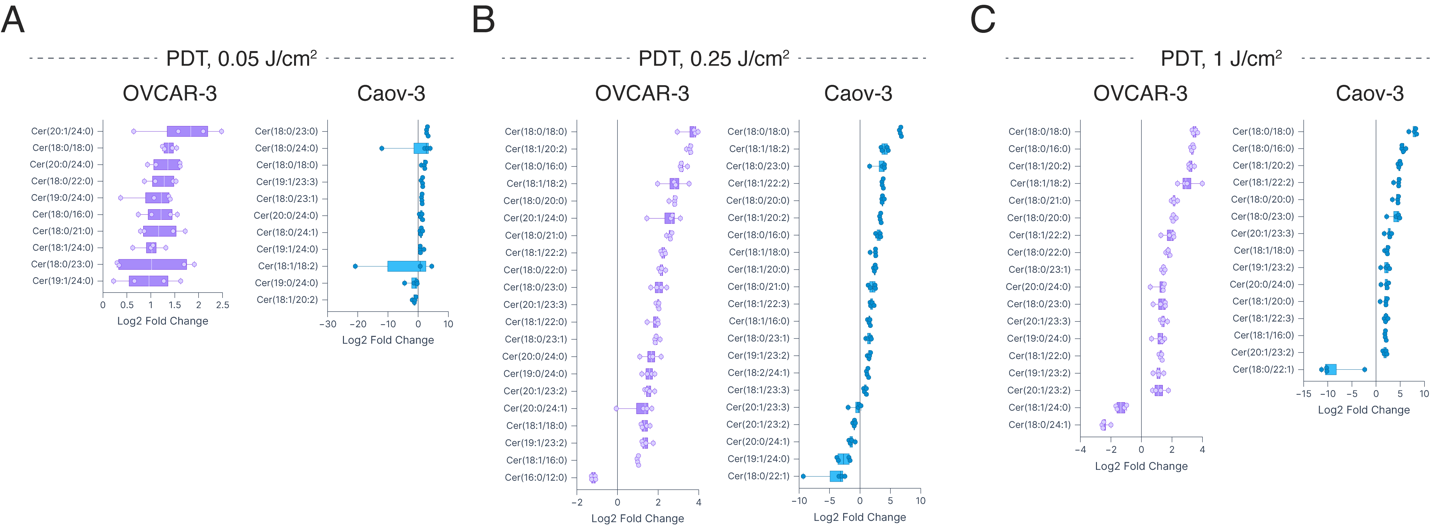


**Figure S24.** Ceramides with absolute log2 fold change > 1 and p value <0.05 in OVCAR-3 and Caov-3 cells treated with PDT at **(A)** 0.05, **(B)** 0.25, and **(C)** 1 J/cm^2^. Each point represents an independent biological replicate, whiskers represent the range. Plots were generated in Graphmatik (Version 0.3.2, 2025).


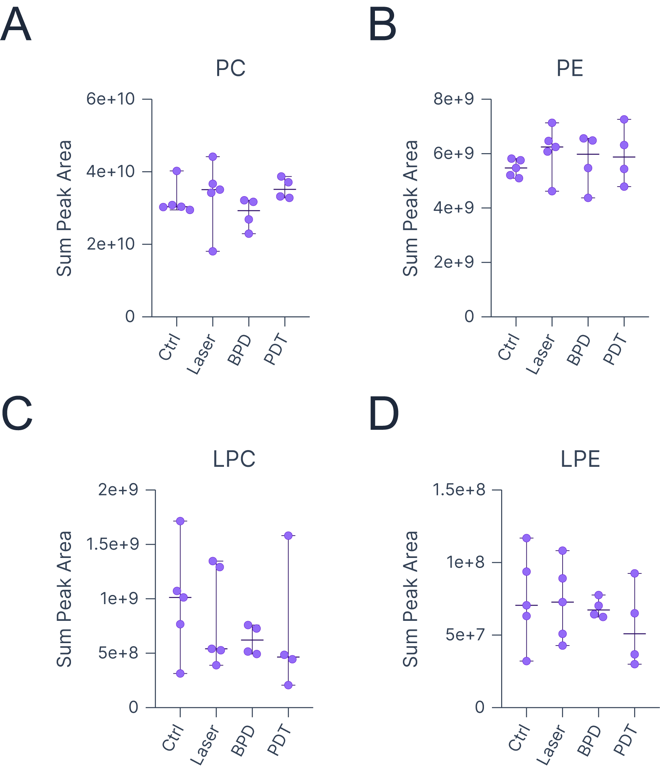


**Figure S25.** Class-level lipidome differences in OVCAR-3 tumors. Each point represents the sum peak area of all lipids within the respective class within a tumor sample. Error bars represent the range. All graphs were generated in Graphmatik (ver. 0.3.2, 2025).

**Table S1.** Four parameter nonlinear regression fit of Cum OOH dose-responses in OVCAR-3 and Caov-3 cells (Graphmatik v. 0.3.2).

|  | OVCAR-3 | | | Caov-3 | | |
| --- | --- | --- | --- | --- | --- | --- |
|  | 10 k/well | 5 k/well | 2.5 k/well | 10 k/well | 5 k/well | 2.5 k/well |
|  | (x, y) | (x, y) | (x, y) | (x, y) | (x, y) | (x, y) |
| Num of Samples | 8 | 8 | 8 | 8 | 8 | 8 |
| Mean | 249,0.28105 | 249,0.25456 | 249,0.22975 | 249,0.67518 | 249,0.54507 | 249,0.40787 |
|  |  |  |  |  |  |  |
| Std. Deviation | 345.83,0.40542 | 345.83,0.39863 | 345.83,0.32465 | 345.83,0.43943 | 345.83,0.4758 | 345.83,0.40421 |
| Std. Error of Mean | 122.27,0.14334 | 122.27,0.14094 | 122.27,0.11478 | 122.27,0.15536 | 122.27,0.16822 | 122.27,0.14291 |
|  |  |  |  |  |  |  |
| Parameters: |  |  |  |  |  |  |
| Bottom | 0.003691 | −3.975e-5 | −6.435e-5 | −0.001558 | −0.000214 | 0.002513 |
| Top | 0.98852 | 1.086 | 2.022 | 1.083 | 1.1025 | 0.90064 |
| β1 | 6.9216 | 5.46 | 2.5507 | 3.8272 | 4.076 | 4.2349 |
| EC or IC50 | 25.601 | 21.332 | 5.9571 | 200.56 | 94.546 | 67.991 |
|  |  |  |  |  |  |  |
| Weighted SSR | 0.003539 | 0.01183 | 0.0122 | 0.01217 | 0.01091 | 0.008626 |
| R squared | 0.99692 | 0.98936 | 0.98347 | 0.991 | 0.99312 | 0.99246 |

**Table S2.** List of differentially expressed (absolute log2 fold change >1, p value < 0.05) lipids between OVCAR-3 and Caov-3 cells.

| LipidID | Fold Change,  Caov-3/OVCAR-3 | log2 FC,  Caov-3/OVCAR-3 | P Value |
| --- | --- | --- | --- |
| AcCa(14:0)+H | 8.59249003 | 3.10307627 | 0.02255995 |
| AcCa(15:0)+H | 14.7841963 | 3.88598391 | 0.01836632 |
| AcCa(16:0)+H | 34.5734633 | 5.11159323 | 0.00142254 |
| AcCa(16:1)+H | 16.8806923 | 4.07730217 | 0.01415153 |
| AcCa(17:0)+H | 27.7224454 | 4.79298262 | 9.9319E-05 |
| AcCa(17:1)+H | 25.9562822 | 4.69801185 | 0.00729881 |
| AcCa(18:0)+H | 157.687471 | 7.30092423 | 0.00078198 |
| AcCa(18:1)+H | 65.5491104 | 6.03450429 | 0.00627298 |
| AcCa(18:2)+H | 36.8906623 | 5.20518379 | 0.01187502 |
| AcCa(19:0)+H | 255.168482 | 7.99530633 | 0.00086291 |
| AcCa(19:1)+H | 316.992583 | 8.30830527 | 0.00089022 |
| AcCa(20:0)+H | 186.744847 | 7.54492463 | 0.0026716 |
| AcCa(20:1)+H | 542.915014 | 9.08458257 | 0.02348163 |
| AcCa(20:2)+H | 1819.58209 | 10.8293914 | 0.00835303 |
| AcCa(20:3)+H | 37.3795258 | 5.22417636 | 0.00440871 |
| AcCa(20:4)+H | 14.4124626 | 3.84924496 | 0.01035898 |
| AcCa(21:0)+H | 940.885793 | 9.87787581 | 0.01091417 |
| AcCa(22:0)+H | 763.345096 | 9.57619161 | 0.00298976 |
| AcCa(22:1)+H | 1702.02483 | 10.7330364 | 0.00399246 |
| AcCa(22:4)+H | 127.14002 | 6.99027441 | 0.0004875 |
| AcCa(22:5)+H | 40.2755787 | 5.33183341 | 0.00764041 |
| AcCa(24:0)+H | 1269.79525 | 10.3103802 | 0.00152217 |
| AcCa(24:1)+H | 22.0659466 | 4.46374973 | 0.00126775 |
| AcCa(24:2)+H | 1724911508 | 30.6838752 | 0.01887612 |
| AcCa(24:3)+H | 145269495 | 27.1141565 | 0.01799244 |
| AcCa(25:1)+H | 2793062.27 | 21.4134163 | 0.02683038 |
| AcCa(26:1)+H | 1742.76635 | 10.7671634 | 0.00314505 |
| Cer(d18:0_16:0)+H | 0.25182272 | -1.9895196 | 0.00172591 |
| Cer(d18:0_18:0)+H | 0.0137629 | -6.1830716 | 0.00011328 |
| Cer(d18:0_20:0)+H | 0.05142022 | -4.2815203 | 0.00194549 |
| Cer(d18:0_21:0)+H | 0.07849955 | -3.6711719 | 0.02734235 |
| Cer(d18:0_22:1)+H | 0.10127109 | -3.3037057 | 0.00161835 |
| Cer(d18:0_23:0)+H | 0.13182698 | -2.9232824 | 0.00189102 |
| Cer(d18:0_23:1)+H | 0.26264916 | -1.9287911 | 0.00214837 |
| Cer(d18:0_24:1)+H | 0.07784762 | -3.6832033 | 0.00467945 |
| Cer(d18:1_16:0)+H | 2.03363319 | 1.02405948 | 0.0102896 |
| Cer(d18:1_18:0)+H | 0.38837936 | -1.3644615 | 8.3436E-05 |
| Cer(d18:1_20:0)+H | 0.18427482 | -2.4400691 | 0.00070089 |
| Cer(d18:1_20:2)+H | 0.04085534 | -4.6133316 | 0.00263465 |
| Cer(d18:1_22:0)+H | 0.38709083 | -1.369256 | 0.00298874 |
| Cer(d18:1_22:2)+H | 0.05910682 | -4.0805316 | 0.00229686 |
| Cer(d18:1_22:3)+H | 0.26767171 | -1.9014634 | 0.01022092 |
| Cer(d19:0_24:0)+H | 0.23582047 | -2.0842391 | 0.00223667 |
| Cer(d19:1_23:2)+H | 0.08613778 | -3.5372101 | 0.00168698 |
| Cer(d19:1_24:0)+H | 0.16758233 | -2.577058 | 0.04378471 |
| Cer(d20:0_24:0)+H | 0.13950627 | -2.8415981 | 7.3523E-05 |
| Cer(d20:0_24:1)+H | 0.15003336 | -2.7366448 | 0.02323106 |
| Cer(d20:1_23:2)+H | 0.15930541 | -2.6501328 | 0.01093855 |
| Cer(d20:1_23:3)+H | 0.11819888 | -3.0807118 | 0.00067845 |
| CL(16:1_16:1_18:2_18:2)-H | 0.12928426 | -2.9513815 | 0.02623 |
| CL(18:1_18:1_18:1_18:1)-H | 4.29572946 | 2.10290314 | 0.0021404 |
| CL(72:4)-H | 4.29572946 | 2.10290314 | 0.0021404 |
| DG(12:1_29:5)+H | 5.08850348 | 2.34724143 | 0.00450712 |
| DG(O-16:3_22:2)+H | 2.49013961 | 1.31622663 | 0.00948964 |
| Hex1Cer(d18:1_18:0)+H | 0.13298998 | -2.9106105 | 2.8899E-05 |
| Hex1Cer(d18:1_20:0)+H | 0.11694134 | -3.0961431 | 0.00058199 |
| Hex1Cer(d18:1_22:0)+H | 0.24782917 | -2.0125821 | 0.0006649 |
| Hex1Cer(d18:1_23:3)+H | 0.28824447 | -1.7946351 | 0.00411734 |
| Hex2Cer(d18:1_18:0)+H | 0.10929429 | -3.19371 | 0.00012415 |
| Hex2Cer(d18:1_20:0)+H | 0.01449684 | -6.108118 | 0.00159056 |
| Hex2Cer(d18:1_22:0)+H | 0.02508974 | -5.3167586 | 0.00368082 |
| Hex2Cer(d18:1_22:1)+H | 0.0717564 | -3.8007488 | 0.00970204 |
| Hex2Cer(d18:1_22:2)+H | 0.04102808 | -4.6072445 | 0.00140679 |
| Hex2Cer(d18:1_23:0)+H | 0.06208413 | -4.0096317 | 0.00110935 |
| Hex2Cer(d18:1_24:0)+H | 0.10289429 | -3.2807651 | 0.03409753 |
| Hex2Cer(d18:1_24:1)+H | 0.11674836 | -3.0985258 | 0.02451136 |
| Hex2Cer(d19:1_23:2)+H | 0.14628756 | -2.7731211 | 0.00233423 |
| Hex2Cer(d20:1_24:1)+H | 0.11569468 | -3.1116055 | 0.03617178 |
| Hex3Cer(d18:1_16:0)+H | 46.6491621 | 5.54377926 | 0.0026912 |
| Hex3Cer(d18:1_24:1)+H | 14.7725909 | 3.88485097 | 0.04584534 |
| LPC(14:0)+H | 0.21935831 | -2.1886388 | 0.01360202 |
| LPC(16:0)+H | 0.35855117 | -1.4797491 | 0.00257287 |
| LPC(18:0)+H | 0.39204152 | -1.3509217 | 0.00071605 |
| LPC(18:1)+H | 0.45552485 | -1.1343983 | 0.0305696 |
| LPC(19:0)+H | 0.39653815 | -1.3344684 | 0.03863262 |
| LPC(20:1)+H | 0.4325411 | -1.2090909 | 0.00160385 |
| LPC(20:4)+H | 0.49762173 | -1.0068786 | 0.0279705 |
| LPC(24:1)+H | 2.46000306 | 1.29866011 | 0.02885193 |
| LPC(30:0)+H | 3.65718838 | 1.87073494 | 0.000429 |
| LPC(30:1)+H | 2.06581218 | 1.04670909 | 0.00010201 |
| LPC(30:2)+H | 2.00072131 | 1.00052022 | 5.4374E-05 |
| LPC(30:3)+H | 0.30550083 | -1.7107518 | 3.4033E-05 |
| LPC(30:4)+H | 0.29892242 | -1.742157 | 0.00744336 |
| LPC(O-18:2)+H | 0.44033647 | -1.1833218 | 0.0133287 |
| LPC(P-15:0)+H | 9.37000127 | 3.22804924 | 0.00147971 |
| LPC(P-16:0)+H | 2.24827853 | 1.16882078 | 0.00125906 |
| LPC(P-17:0)+H | 3.92048646 | 1.97103268 | 0.00028275 |
| LPC(P-18:0)+H | 4.8153515 | 2.26764111 | 0.04462682 |
| LPC(P-20:0)+H | 5.68570433 | 2.50733908 | 0.01605453 |
| LPC(P-20:3)+H | 4.03898139 | 2.0139915 | 0.0263531 |
| LPC(P-22:0)+H | 2.5231593 | 1.3352313 | 0.01260991 |
| LPC(P-24:0)+H | 5.34105953 | 2.41712596 | 0.01779413 |
| LPE(18:0)+H | 0.36538807 | -1.4524986 | 0.02088923 |
| PC(17:2_17:2)+H | 0.30045351 | -1.7347863 | 5.9506E-07 |
| PC(22:3_18:1)+H | 9.84763037 | 3.29977661 | 0.00022289 |
| PC(22:6_18:0)+H | 2.03044607 | 1.02179671 | 0.00267934 |
| PC(23:7_22:4)+H | 49.6549044 | 5.63386431 | 0.04005132 |
| PC(32:2)+H | 0.37964971 | -1.3972592 | 0.00736167 |
| PC(32:4)+H | 0.05811128 | -4.105038 | 0.00590636 |
| PC(32:5)+H | 0.19162499 | -2.3836424 | 0.03103491 |
| PC(34:3)+H | 0.49819495 | -1.0052177 | 0.00010553 |
| PC(34:5)+H | 0.26679591 | -1.9061915 | 0.01052667 |
| PC(34:6)+H | 0.19102721 | -2.3881499 | 0.02207525 |
| PC(34:7)+H | 0.09867156 | -3.3412219 | 0.00174718 |
| PC(35:3)+H | 0.38382844 | -1.3814665 | 0.00025665 |
| PC(35:7)+H | 0.32830303 | -1.6069 | 0.00526576 |
| PC(36:5)+H | 0.44384449 | -1.1718738 | 0.00726308 |
| PC(36:6)+H | 0.2207763 | -2.1793428 | 0.01396623 |
| PC(36:7)+H | 0.44832426 | -1.1573855 | 0.00053994 |
| PC(36:8)+H | 0.23708921 | -2.0764981 | 0.00758575 |
| PC(37:6)+H | 0.37927228 | -1.3986942 | 0.00133337 |
| PC(38:7)+H | 0.37060802 | -1.432034 | 0.0138813 |
| PC(38:8)+H | 0.23755064 | -2.073693 | 0.01973547 |
| PC(38:9)+H | 0.34834454 | -1.5214131 | 0.01566863 |
| PC(40:10)+H | 0.305542 | -1.7105574 | 0.01631511 |
| PC(40:3)+H | 2.93907191 | 1.55536066 | 0.00143305 |
| PC(41:2)+H | 2.06633787 | 1.04707617 | 0.0337543 |
| PC(41:4)+H | 7.41143166 | 2.88975225 | 0.00091466 |
| PC(43:7)+H | 7.71022987 | 2.94677387 | 0.00213617 |
| PC(44:5)+H | 2.14221901 | 1.09910598 | 0.00089922 |
| PC(48:5)+H | 0.07659485 | -3.7066088 | 0.0020615 |
| PC(50:6)+H | 0.19776762 | -2.3381218 | 0.00504086 |
| PC(50:7)+H | 6.56807518 | 2.71547064 | 0.00183462 |
| PC(50:8)+H | 2.28768681 | 1.19388956 | 0.00108075 |
| PC(O-10:0_20:0)+H | 3.70804994 | 1.89066067 | 0.00034173 |
| PC(O-15:1_16:0)+H | 0.45816421 | -1.1260633 | 0.02694355 |
| PC(O-15:1_18:1)+H | 0.48160506 | -1.0540775 | 0.04382301 |
| PC(O-16:1_20:3)+H | 2.44701509 | 1.291023 | 0.00248411 |
| PC(O-18:3_20:2)+H | 3.15058082 | 1.65561782 | 1.1478E-05 |
| PC(O-18:4_16:1)+H | 2.32143148 | 1.2150147 | 0.00139457 |
| PC(O-18:4_18:0)+H | 2.91971758 | 1.54582883 | 0.00090575 |
| PC(O-31:0)+H | 4.87733477 | 2.286093 | 0.00133059 |
| PC(O-31:1)+H | 2.89491017 | 1.53351858 | 0.00704004 |
| PC(O-31:3)+H | 0.07729214 | -3.6935345 | 0.01396803 |
| PC(O-32:0)+H | 3.14896292 | 1.65487677 | 0.0002696 |
| PC(O-32:1)+H | 2.74431818 | 1.45644776 | 8.0973E-05 |
| PC(O-32:5)+H | 0.14794256 | -2.756891 | 0.00258516 |
| PC(O-33:0)+H | 4.60258405 | 2.20244407 | 0.00158065 |
| PC(O-33:1)+H | 2.97537543 | 1.57307172 | 0.00055909 |
| PC(O-33:2)+H | 2.39768136 | 1.26163994 | 0.00128939 |
| PC(O-33:6)+H | 0.31220495 | -1.6794347 | 0.04981468 |
| PC(O-34:1)+H | 2.81832003 | 1.49483544 | 0.00015572 |
| PC(O-34:4)+H | 2.55970193 | 1.35597582 | 0.0015432 |
| PC(O-34:5)+H | 0.44191076 | -1.178173 | 0.00135258 |
| PC(O-34:6)+H | 0.32355535 | -1.6279156 | 0.00047633 |
| PC(O-34:7)+H | 0.32578029 | -1.6180288 | 0.01313719 |
| PC(O-35:0)+H | 4.11911342 | 2.04233385 | 0.00184577 |
| PC(O-35:1)+H | 3.24712803 | 1.69916427 | 0.00488585 |
| PC(O-35:4)+H | 4.20206612 | 2.07109886 | 0.02081248 |
| PC(O-36:0)+H | 2.47911276 | 1.30982389 | 0.0358938 |
| PC(O-36:1)+H | 4.6989922 | 2.23235137 | 0.00142886 |
| PC(O-36:2)+H | 4.15463918 | 2.05472319 | 0.00301009 |
| PC(O-36:3)+H | 2.62364294 | 1.39157139 | 0.00017743 |
| PC(O-36:4)+H | 2.45461365 | 1.29549597 | 0.00124201 |
| PC(O-36:5)+H | 2.87718178 | 1.52465637 | 0.00037275 |
| PC(O-36:6)+H | 2.42707219 | 1.27921702 | 0.00161918 |
| PC(O-36:7)+H | 2.14415812 | 1.1004113 | 7.9388E-05 |
| PC(O-36:8)+H | 2.36208581 | 1.24006138 | 0.00832643 |
| PC(O-37:4)+H | 3.34676997 | 1.7427694 | 0.00708493 |
| PC(O-38:1)+H | 5.80082721 | 2.53625865 | 0.02791171 |
| PC(O-38:2)+H | 7.24781933 | 2.85754699 | 0.02760756 |
| PC(O-38:3)+H | 5.81093128 | 2.53876939 | 0.00369641 |
| PC(O-38:4)+H | 2.996633 | 1.58334241 | 0.00921882 |
| PC(O-40:2)+H | 20.1084529 | 4.32973018 | 0.03066787 |
| PC(O-40:3)+H | 5.18927442 | 2.37553283 | 0.00222691 |
| PC(O-40:4)+H | 5.95537752 | 2.57419296 | 0.00721138 |
| PC(O-40:5)+H | 3.30728839 | 1.72564885 | 0.00014324 |
| PC(O-40:7)+H | 3.35973818 | 1.74834881 | 0.02991308 |
| PC(O-40:8)+H | 2.45543485 | 1.29597854 | 0.00026383 |
| PC(O-41:1)+H | 4.54667396 | 2.18481155 | 0.01568942 |
| PC(O-42:2)+H | 2.81903007 | 1.49519887 | 0.039034 |
| PC(O-42:3)+H | 5.28704948 | 2.40246283 | 0.04535446 |
| PC(O-42:5)+H | 4.23544555 | 2.08251374 | 0.00613195 |
| PC(O-42:6)+H | 3.82363254 | 1.93494388 | 0.0077941 |
| PC(O-42:7)+H | 44.8800025 | 5.48800085 | 0.01667584 |
| PC(O-44:6)+H | 2.60580906 | 1.38173137 | 0.00945237 |
| PC(O-44:7)+H | 4.29612866 | 2.1030372 | 0.03551241 |
| PC(O-46:7)+H | 4.07195502 | 2.02572163 | 0.00529187 |
| PC(O-48:8)+H | 0.02632272 | -5.2475476 | 0.01488388 |
| PC(P-18:4_16:0)+H | 2.49204053 | 1.31732753 | 0.00056748 |
| PC(P-18:4_18:1)+H | 2.45373347 | 1.29497855 | 0.00252162 |
| PC(P-31:7)+H | 0.14837209 | -2.7527083 | 0.00375122 |
| PC(P-32:0)+H | 2.40447008 | 1.26571897 | 3.8649E-05 |
| PE(14:0_26:6)-H | 0.37939091 | -1.398243 | 0.00092594 |
| PE(24:3_18:0)-H | 6.82380336 | 2.77057607 | 0.01410996 |
| PE(P-12:0_16:0)+H | 0.08381201 | -3.5766992 | 0.00371152 |
| PE(P-12:0_20:4)+H | 0.1267779 | -2.9796248 | 0.00158899 |
| PE(P-12:0_22:6)+H | 0.09644294 | -3.3741806 | 0.00014558 |
| PE(P-16:0_16:0)+H | 0.39717068 | -1.332169 | 0.00952948 |
| PE(P-16:0_16:1)+H | 0.3182629 | -1.6517091 | 0.00566604 |
| PE(P-18:0_16:0)+H | 0.47093474 | -1.0864009 | 0.02562971 |
| PE(P-18:0_18:1)+H | 0.49641336 | -1.0103862 | 0.04717974 |
| PE(P-18:1_16:1)+H | 0.48115805 | -1.0554172 | 0.00067276 |
| PE(P-18:1_20:5)+H | 2.04793315 | 1.03416862 | 0.01165727 |
| PG(18:4_20:1)-H | 0.11150818 | -3.1647786 | 6.9189E-05 |
| PG(20:1_13:0)-H | 0.18543367 | -2.4310249 | 1.0461E-05 |
| PG(20:4_22:6)+H | 0.24133303 | -2.0509027 | 0.00327217 |
| PG(22:6_18:1)+H | 0.48830811 | -1.0341364 | 0.00014038 |
| PI(O-27:0_23:7)-H | 0.13401923 | -2.8994881 | 0.00506329 |
| PS(12:0_23:2)-H | 0.33718201 | -1.5684005 | 0.00156172 |
| PS(30:3_14:0)-H | 0.15921747 | -2.6509294 | 0.00010707 |
| SM(d18:1_19:3)+H | 4.50212462 | 2.17060599 | 0.00553356 |
| SM(d18:1_24:2)+H | 0.06009002 | -4.0567309 | 0.00161411 |
| SM(d18:1_26:3)+H | 0.22141691 | -2.1751627 | 9.9816E-05 |
| SM(d30:0)+H | 0.02880682 | -5.117446 | 0.00876757 |
| SM(d30:1)+H | 0.29648826 | -1.7539531 | 0.04521573 |
| SM(d32:0)+H | 0.1171034 | -3.0941451 | 0.00486703 |
| SM(d34:0)+H | 0.31022222 | -1.6886261 | 8.7904E-06 |
| SM(d34:1)+H | 2.38909829 | 1.25646621 | 0.00083876 |
| SM(d34:2)+H | 2.70464273 | 1.43543804 | 0.00243912 |
| SM(d34:3)+H | 0.15927567 | -2.6504022 | 1.761E-07 |
| SM(d36:0)+H | 0.03606515 | -4.7932508 | 3.9335E-05 |
| SM(d36:1)+H | 0.3058094 | -1.7092953 | 7.1963E-07 |
| SM(d36:3)+H | 0.31696393 | -1.6576094 | 0.01892164 |
| SM(d36:4)+H | 2.84653309 | 1.50920587 | 0.01720456 |
| SM(d36:5)+H | 5.62976203 | 2.49307394 | 0.00050096 |
| SM(d37:1)+H | 0.08567304 | -3.545015 | 0.00027029 |
| SM(d38:0)+H | 0.03833645 | -4.7051395 | 0.00227195 |
| SM(d38:1)+H | 0.17421603 | -2.5210507 | 0.00036714 |
| SM(d38:2)+H | 0.40347292 | -1.3094563 | 3.0193E-05 |
| SM(d38:4)+H | 0.23881883 | -2.0660115 | 0.02661118 |
| SM(d39:0)+H | 0.00299698 | -8.3822752 | 0.00129772 |
| SM(d40:0)+H | 0.05322202 | -4.231833 | 0.00175527 |
| SM(d40:1)+H | 0.47109267 | -1.0859172 | 0.00564625 |
| SM(d40:3)+H | 0.0150555 | -6.0535656 | 0.00020886 |
| SM(d40:4)+H | 0.12034258 | -3.0547809 | 0.00204521 |
| SM(d41:0)+H | 0.0615322 | -4.0225147 | 0.00903079 |
| SM(d42:3)+H | 2.43279314 | 1.28261365 | 0.0103943 |
| SM(d42:4)+H | 0.47989484 | -1.0592098 | 0.00702616 |
| SM(d43:3)+H | 0.01406843 | -6.1513953 | 0.00423132 |
| SM(d44:1)+H | 0.18383799 | -2.4434932 | 0.00185204 |
| SM(d44:5)+H | 0.24941715 | -2.0033674 | 1.5778E-06 |
| SM(d44:6)+H | 2.58654174 | 1.37102447 | 1.6423E-05 |
| SPH(d18:1)+H | 0.23477366 | -2.0906575 | 2.3429E-05 |
| SPH(d19:0)+H | 0.0177963 | -5.8122788 | 0.00373143 |
| TG(18:1_18:1_18:1)+H | 2.04827556 | 1.03440982 | 0.03355063 |
| TG(18:1_18:1_22:3)+H | 2.02420646 | 1.01735645 | 0.02133156 |
| TG(43:2_19:4)+H | 2.5147207 | 1.33039817 | 0.04698616 |
| TG(O-16:0_19:2_19:2)+H | 3.42888131 | 1.77773797 | 0.0114531 |
| TG(O-30:4_11:0_11:0)+H | 4.24741369 | 2.08658463 | 0.01417444 |
| TG(O-60:12_3:0)+H | 2.38245991 | 1.25245194 | 0.00951622 |

**Table S3.** Four parameter nonlinear regression fit of ML210 dose-responses in OVCAR-3 and Caov-3 cells (Graphmatik v. 0.3.2).

|  | OVCAR-3 | | | Caov-3 | | |
| --- | --- | --- | --- | --- | --- | --- |
|  | 10 k/well | 5 k/well | 2.5 k/well | 10 k/well | 5 k/well | 2.5 k/well |
|  | (x, y) | (x, y) | (x, y) | (x, y) | (x, y) | (x, y) |
| Num of Samples | 7 | 7 | 7 | 7 | 7 | 7 |
| Mean | 2.8348,0.47806 | 2.8348,0.40919 | 2.8348,0.36954 | 2.8348,0.89037 | 2.8348,0.79133 | 2.8348,0.70492 |
|  |  |  |  |  |  |  |
| Std. Deviation | 3.584,0.39512 | 3.584,0.36095 | 3.584,0.33571 | 3.584,0.13437 | 3.584,0.17731 | 3.584,0.22245 |
| Std. Error of Mean | 1.3546,0.14934 | 1.3546,0.13643 | 1.3546,0.12689 | 1.3546,0.05079 | 1.3546,0.06702 | 1.3546,0.08408 |
|  |  |  |  |  |  |  |
| Parameters: |  |  |  |  |  |  |
| Bottom | −0.003475 | −0.002835 | −0.003373 | 0.5 | 0.49965 | 0.39887 |
| Top | 1.085 | 1.1215 | 1.2126 | 1 | 1.086 | 1.1809 |
| β1 | 2.9976 | 2.5202 | 2.4082 | 2.9126 | 2.4417 | 2.3194 |
| EC or IC50 | 1.013 | 0.68439 | 0.39414 | 5.7172 | 1.114 | 0.62986 |
|  |  |  |  |  |  |  |
| Weighted SSR | 0.004937 | 0.01076 | 0.002262 | 0.002708 | 0.006829 | 0.008074 |
| R squared | 0.99473 | 0.98624 | 0.99666 | 0.975 | 0.9638 | 0.97281 |
